# IMA Genome – F19: A genome assembly and annotation guide to empower mycologists, including annotated draft genome sequences of *Ceratocystis pirilliformis*, *Diaporthe australafricana*, *Fusarium ophioides*, *Paecilomyces lecythidis*, and *Sporothrix stenoceras*

**DOI:** 10.1186/s43008-024-00142-z

**Published:** 2024-06-03

**Authors:** Janneke Aylward, Andi M. Wilson, Cobus M. Visagie, Joseph Spraker, Irene Barnes, Carla Buitendag, Callin Ceriani, Lina Del Mar Angel, Deanné du Plessis, Taygen Fuchs, Katharina Gasser, Daniella Krämer, WenWen Li, Kiara Munsamy, Anja Piso, Jenna-Lee Price, Byron Sonnekus, Chanel Thomas, Ariska van der Nest, Alida van Dijk, Alishia van Heerden, Nicole van Vuuren, Neriman Yilmaz, Tuan A. Duong, Nicolaas A. van der Merwe, Michael J. Wingfield, Brenda D. Wingfield

**Affiliations:** 1 Department of Biochemistry, Genetics and Microbiology, Forestry and Agricultural Biotechnology Institute (FABI), University of Pretoria, Pretoria, 0028 South Africa; 2 Department of Conservation Ecology and Entomology, Stellenbosch University, Private Bag X1, Matieland, 7602 South Africa; 3 Hexagon Bio, 1490 O’Brien Dr, Menlo Park, CA 94025 USA; 4 Department of Crop Sciences, University of Natural Resources and Life Sciences (BOKU), Institute of Plant Protection, Konrad Lorenz-Strasse 24, Tulln an Der Donau, 3430 Vienna, Austria; 5 Department of Plant and Soil Sciences, Forestry and Agricultural Biotechnology Institute (FABI), University of Pretoria, Pretoria, 0028 South Africa

## Abstract

The pace at which Next Generation Sequence data is being produced continues to accelerate as technology improves. As a result, such data are increasingly becoming accessible to biologists outside of the field of bioinformatics. In contrast, access to training in the methods of genome assembly and annotation are not growing at a similar rate. In this issue, we report on a Genome Assembly Workshop for Mycologists that was held at the Forestry and Agricultural Biotechnology Institute (FABI) at the University of Pretoria, South Africa and make available the 12 draft genome sequences emanating from the event. With the aim of making the process of genome assembly and annotation more accessible to biologists, we provide a step-by-step guide to both genome assembly and annotation, intended to encourage and empower mycologists to use genome data in their research.

**Supplementary Information:**

The online version contains supplementary material available at 10.1186/s43008-024-00142-z.

## Introduction

Next Generation Sequencing (NGS) technologies have had a transformative impact on biological research (McGuire et al. 2020). The availability of whole genome data has enabled the identification of genes and genetic markers, offering opportunities to unravel complex biological processes at individual, cellular, and population levels. Despite the obvious importance of NGS, it remains under-utilised in many instances (Williams and Teal 2017; Batut et al. 2018). This is partly due to the multidisciplinary nature of such research, which requires expertise in both biological and computational sciences (Magana et al. 2014). Biologists performing these analyses often require additional training to use the available sophisticated computational tools that can handle the volume and complexity of their data (Batut et al. 2018), while computer scientists may require guidance regarding the biological context and interpretation of the data.

As technology improves, our ability to generate complex NGS datasets continues to grow. It is, consequently, imperative that researchers become skilled in the use of NGS data, and that infrastructure to provide bioinformatics training to biologists grows at a similar pace (Williams and Teal 2017). Unfortunately, this is not always the case. To address this need, a week-long Genome Assembly Workshop for Mycologists was held at the Forestry and Agricultural Biotechnology Institute (FABI) at the University of Pretoria in South Africa, in July 2023. The course was arranged and led by the first authors of this paper and attended by academic staff, postdoctoral researchers, and graduate students. All attendees had a background in mycology and most had little or no experience with genomic analyses and the command-line environment.

The aims of the workshop were to guide participants through UNIX-based bioinformatics analyses and to produce annotated genome assemblies that could be made publicly available. For this purpose, the practical sessions provided the opportunity for the participants to assemble and annotate a set of fungal genomes under the supervision of the workshop leaders. These steps, which were followed for all genome assemblies presented in this edition of IMA Genome, are summarised in the Methods section. They have also been compiled into a step-by-step guide, which is available here as supplementary documentation (File S1). It is important to recognize that this guide is intended to illustrate the general flow of a genome assembly and annotation project, largely employing scripts that beginners should be able to use, given access to a suitable computing platform. For every step in this guide, numerous alternative programs are available, and we do not claim that those used here are superior. By providing this guide, we hope to make the process of genome assembly and annotation more accessible to biologists with little computational experience. In addition to the training outcomes of this workshop, we also present 12 draft genomes from five species of fungi relevant to agriculture and forestry.

## Methods

### Fungal isolates

The sequence data of five filamentous ascomycete fungi used to train the workshop participants were provided through a collaboration between FABI (https://www.fabinet.up.ac.za) and Hexagon Bio (https://www.hexagonbio.com). The species included two strains of *Ceratocystis pirilliformis* (CMW-IA:5519, CMW-IA:4944), *Diaporthe australafricana* (CMW-IA:644, CMW-IA:616) and *Paecilomyces lecythidis* (CMW-IA:5347, CMW-IA:5364) and three strains of *Fusarium ophioides* (CMW-IA:5007, CMW-IA:5006, CMW-IA:4746) and *Sporothrix stenoceras* (CMW-IA:5313, CMW-IA:5347, CMW-IA:5364). These ascomycete fungi were chosen based on the small size of their genomes (20–50 Mb) and their relevance to the research of the participants. All isolates are stored in the culture collections (CMW and CMW-IA) of the Forestry and Agricultural Biotechnology Institute (FABI) at the University of Pretoria.

### DNA extraction and sequencing

All isolates were grown on malt and yeast extract (MEYA) medium consisting of 2% w/v malt extract, 0.5% w/v yeast extract and 0.5% w/v agar at 25 °C for 14 days. Mycelium was harvested, frozen at -80 °C, lyophilized, and pulverised via bead beating. Genomic DNA was purified from ground mycelial powder with a Zymobiomics MagBead DNA/RNA (Zymo Research, United States). For preparation of sequencing libraries, ~ 100 ng of total genomic DNA was processed using the KAPA HyperPlus Kit for PCR-free workflows (Roche, Switzerland), followed by seven rounds of amplification to increase library yields. Sequencing libraries were pooled and size-selected for 300–800 bp fragments using a Qiagen GeneRead Size Selection Kit (Qiagen, Germany). The constructed libraries were sequenced on a NovaSeq Sequencing System (Illumina, USA) to obtain paired-end reads of 2 × 151 bp. The quality of the sequenced libraries was assessed using FastQC v0.11.7 (Babraham Bioinformatics, Babraham Institute, Cambridge, UK). Adapters and low-quality reads were removed using Trimmomatic v0.36 (Bolger et al. 2014).

### Genome assembly

SPAdes v3.15.4 (Bankevich et al. 2012) was used to assemble the trimmed reads. This initial assembly was referred to as assembly v1.0. Contigs of less than 500 bp were filtered out using SeqKit v0.10.1 (Shen et al. 2016) to produce assembly v1.1. QUAST v5.0.2 (Gurevich et al. 2013) and BUSCO v5.3.2 (Manni et al. 2021) were used to determine the genome statistics and evaluate the completeness of assembly v1.1. To assess genome completeness, the fungal_odb10, ascomycota_odb10, and bacteria_odb10 lineages were used for BUSCO (benchmarking universal single copy orthologs) analyses on all 12 genomes. Additionally, the *P. lecythidis* genomes were assessed with the eurotiomycete_odb10 lineage and the remaining genomes with the sordariomycete_odb10 lineage. To determine assembly coverage depth, the trimmed sequence reads were mapped back to assembly v1.1 with Bowtie2 v2.4.1 (Langmead and Salzberg 2012). All commands and parameters used for the bioinformatics analyses are provided in File S1.

### Confirmation of species identity

Maximum-likelihood phylogenetic analyses were conducted to confirm the identity of the sequenced isolates. The relevant DNA barcoding region(s) were extracted from each assembly (v1.1) and reference sequences were obtained from GenBank (Table S1). Phylogenies for *Diaporthe australafricana*, *Fusarium ophioides*, *Paecilomyces lecythidis*, and *Sporothrix stenoceras* were computed on the NGPhylogeny.fr platform (Lemoine et al. 2019), using MAFFT v7.407_1 (Katoh and Standley 2013) for sequence alignment, trimAl v1.4.1 (Capella-Gutiérrez et al. 2009) for alignment curation and PhyML + SMS v1.8.1_1 (Guindon et al. 2010) with 1000 bootstrap replicates for tree inference. Sequence alignments for the *Ceratocystis pirilliformis* phylogeny were retrieved from http://purl.org/phylo/treebase/phylows/study/TB2:S22005 (Barnes et al. 2018) and manually edited in Mega V11.0.13. The phylogeny was produced using the Kimura 2-parameter model using the heuristic search option in Mega V11.0.13. See the figure captions of each phylogeny for details relevant to each species.

The morphological characters of each strain were studied by plating them on growth media typically used to morphologically characterise these genera (Samson et al. 2019; Crous et al. 2009, 2021) and incubating them in the dark at 25 °C for 7 days or until sexual or asexual structures were observed (figure legends include details of the media used). The cultures of *Diaporthe australafrican*a were non-fertile and, therefore, excluded from morphological analysis. Colony images were captured using a Sony alpha 7 III camera and a Sony FE 90 mm f/2.8 Macro G OSS lens (Tokyo, Japan). Microscope images were captured with a Zeiss AXIO Imager.A2 compound and AXIO Zoom.V16 microscope and an AxioCaM 512 colour camera. These are controlled by Zen Blue v 3.2 software (Carl Zeiss CMP, Goettingen, Germany). Helicon Focus v 7.5.4 (HeliconSoft, Kharkiv, Ukraine) was used to stack images with extended depth of field. The photographic plates were prepared in Affinity Photo v 2.2.0 (Serif (Europe)). Some microphotographs were processed with the “Inpainting Brush Tool" without changing areas of scientific importance.

### Assessing non-target taxa

To demonstrate a method of investigating an assembly with presumed contamination, BlobToolKit v4.1.5 (Challis et al. 2020) was used to assess the presence of sequences from non-target taxa. A putative taxonomic origin was assigned to each contig in assembly v1.1 using BLASTn v2.14.0 + (Altschul et al. 1990) and DIAMOND BLASTx v2.1.8.162 (Buchfink et al. 2021). This information, along with the coverage depth and BUSCO results, was used to create and view a taxon-annotated GC-coverage plot of each assembly on the BTK Viewer (https://blobtoolkit.genomehubs.org/view). Using the BTK Viewer, assemblies were filtered to discard contigs < 1000 bp in length and with a coverage depth lower than at least 10% of the coverage of the main assembly “blob” (see Fig. S1-S5 for thresholds applied to each species). Following the principles of a BlobPlot (Kumar et al. 2013), sequences that clustered to form blobs with GC and coverage statistics completely different to those of the target genome were also removed. It is crucial to note that removal of sequences requires discretion, so as not to discard data belonging to the target taxon. Additionally, the predicted taxonomic origin of sequences should be considered. In the case of this workshop, contigs that deviated from both the GC and coverage statistics and that had a putative non-fungal origin were removed. The BTK-filtered assembly was named assembly v2.0. This assembly was finalised using the clean and sort functions of the Funannotate v1.8.15 pipeline (Palmer and Stajich 2020) to ensure that no duplicate contigs were present and to rename the scaffolds in descending order. The final assembly (v2.1) was submitted to GenBank before performing further analyses.

### Repeat masking and gene prediction

RepeatModeler v2.0.1 (Flynn et al. 2020) was used to create a custom library of repeat families for each assembly and these were subsequently used to soft mask assembly v2.1 with RepeatMasker open-4.0.7 (Smit et al. 2013). Genes were predicted in the masked assembly (v3.0) with Funannotate predict, which uses Augustus v3.5.0 (Stanke et al. 2008), GeneMark-ES (Ter-Hovhannisyan et al. 2008), Glimmer v3.0.4 (Delcher et al. 2007), and SNAP (Korf 2004) as *ab initio* predictors. It additionally incorporates protein evidence in the form of BLAST alignments from the UniProt database (The UniProt Consortium 2023) and predicts tRNA genes with tRNAscan-SE v2.0.12 (Lowe and Eddy 1997).

### Functional annotation

Putative functions were assigned to the predicted genes by comparing them against multiple annotation databases. These included running stand-alone versions of InterProScan v5.52–86.0 (Jones et al. 2014; Blum et al. 2021), EggNOG-mapper v2.1.11 (Cantalapiedra et al. 2021), antiSMASH v6.1.1 (Blin et al. 2021) and Phobius v1.01 (Käll et al. 2007). These annotations were merged into a single file by running the Funannotate annotate pipeline which additionally compared predicted proteins against the dbCAN v11.0 (Yin et al. 2012) database of carbohydrate-active enzymes (CAZymes) and the MEROPS v12.0 (Rawlings et al. 2014) protease database. Basic comparative statistics within the five different species, as well as among all assemblies presented in this issue, were calculated using the Funannotate compare script (File S2). The mating-type loci were also identified in each of the genomes, using local BLASTn and tBLASTn searches and the *MAT* gene or protein sequences from closely related species (File S3).

## IMA GENOME-F 19A

### Draft genome assemblies of *Ceratocystis pirilliformis* isolates CMW-IA:5519 and CMW-IA:4944

**Fig. 1:**
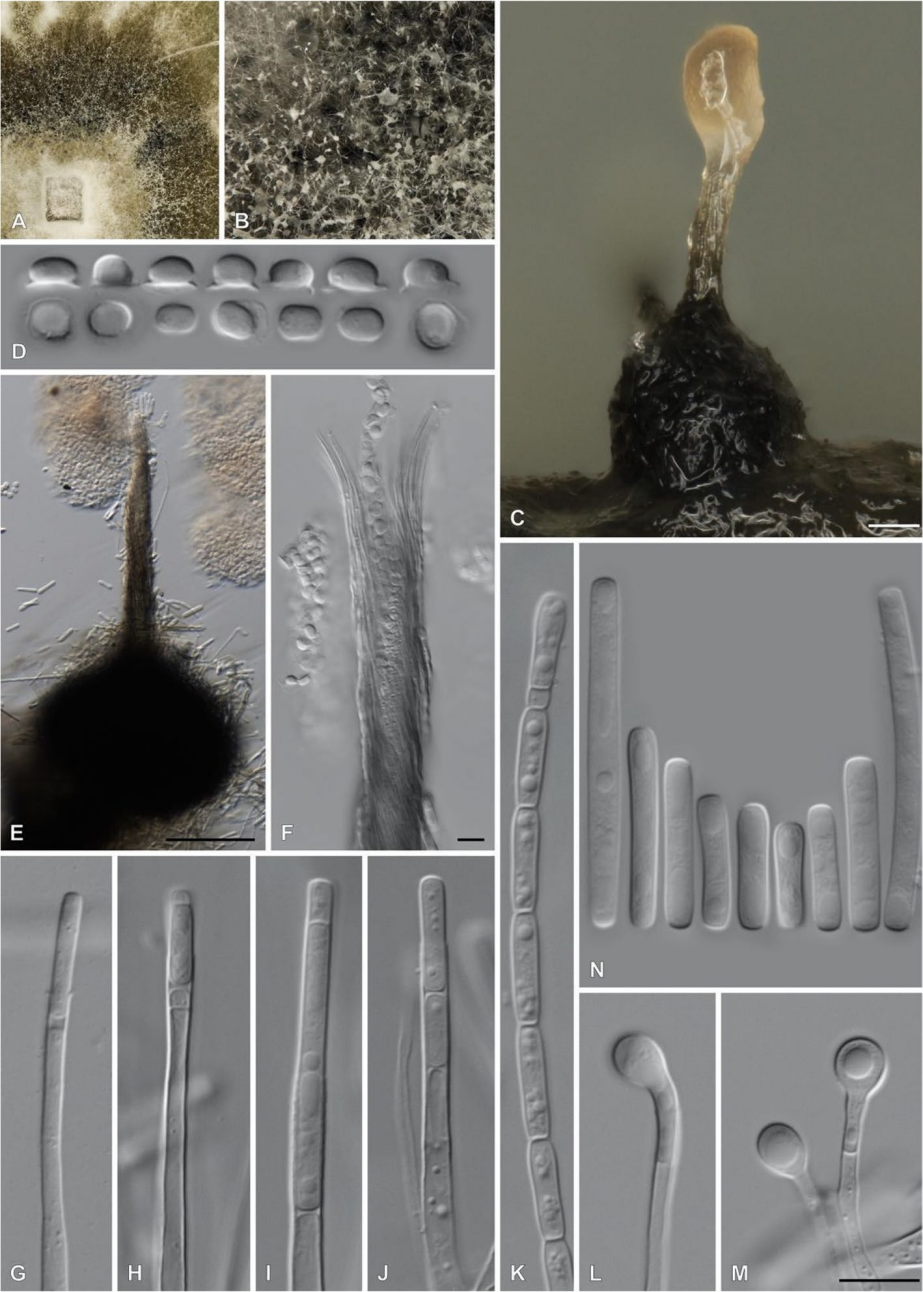
Photoplate of *Ceratocystis pirilliformis* (CMW-IA:5519). **A**, **B**. Close-up of colonies grown for 2 weeks at 25 °C on 2% malt extract agar (MEA) supplemented with streptomycin (150 mg/l) and thiamine (100 mg/l). **C**, **E**. Pear-shaped ascomata. **D**. Hat-shaped ascospores. **F**. Ostiolar hyphae with ascospores emerging through the neck apex. **G**–**K**. Conidiophores. **L**, **M**. Globose to subglobose chlamydospores. **N**. Cylindrical conidia. Bars: **C**, **E** = 100 µm; **F** = 10 µm; M = 10 µm (applies to **D**, **G**–**N**)

The genus *Ceratocystis* in the *Ceratocystidaceae* (order *Microascales*) includes more than 42 species, which all have characteristic perithecial bases (mostly black), with long necks terminating in ostiolar hyphae through which hat-shaped ascospores are released (Fig. 1). The species can be loosely grouped into four phylogenetic clades, roughly characterised by the geographic origin of the species within the clades (Harrington 2000; Mbenoun et al. 2014; Li et al. 2017).

Species in the Latin American Clade (LAC) include some of the best-known and aggressive pathogens important to both agriculture and forestry. They include the type species, *C. fimbriata*, that causes black rot of sweet potato (Marincowitz et al. 2020), *C. manginecans* that has a wide host range but is notorious for having destroyed *Acacia mangium* plantations in South East Asia (Wingfield et al. 2023), *C. eucalypticola* that causes a disease on *Eucalyptus* (Roux et al. 2020), *C. cacaofunesta* that is host specific on cacao (Engelbrecht et al. 2007), and *C. colombiana* that damages coffee plantations in Colombia (van Wyk et al. 2010). Although most of the species in the LAC are considered to be of Latin American origin, many species have been moved extensively around the globe, mostly through anthropogenic means (Oliveira et al. 2015; Liu et al. 2021). The genomes of strains representing all the examples mentioned above have been sequenced (Wilken et al. 2013; van der Nest et al. 2014b; Wingfield et al. 2015, 2022; Molano et al. 2018).

The African Biogeographic Group (African Clade) harbours species that originate or have been described from the African continent, and mostly from native environments and hosts (Roux et al. 2007; Mbenoun et al. 2014). These species are generally not considered serious pathogens. An exception is *C. albifundus* that has caused disease outbreaks on non-native *Acacia mearnsii* trees planted for commercial use in South Africa, and other African countries (Roux and Wingfield 2009). The genome of a strain of *C. albifundus* from *A. mearnsii* in South Africa has been sequenced (van der Nest et al. 2014a).

*Ceratocystis* species in the North American Clade cause disease on a variety of different woody hosts (Holland et al. 2019). Species in this clade for which genomes are available include *C. harringtonii* from poplar (Wingfield et al. 2016), and *C. smalleyi* from hickory (Wingfield et al. 2018). The fourth clade, referred to as the Asian-Australian Clade (AAC), includes species that have been described causing rot on roots and tubers, and colonising the sapwood of trees exposed by wounding (Li et al. 2017; Barnes et al 2018; Liu et al. 2018). Most species are considered to be only mildly pathogenic. There are currently no genome sequences available for any species within this clade.

The first species to be described in the AAC was *C. pirilliformis*; the etymology of the name is derived from the pear-shaped perithecial bases characteristic of this species (Fig. 1; Barnes et al. 2003). It was first discovered in 2003 in Australia, as a wound colonist on *Eucalyptus nitens* (Barnes et al. 2003). It has subsequently been found on Australian species (*A. mearnsii* and several *Eucalyptus* spp.), and on a *Rapanea* species in their native South African environment (Lee et al. 2016). The aim of this study was to sequence the genomes of two strains of *C. pirilliformis* from South Africa as representatives of the AAC for future genomic comparisons among the different clades.

#### Sequenced strains

*Ceratocystis pirilliformis*: South Africa: *Mpumalanga:* Bushbuckridge*,* isolated from *Eucalyptus grandis* × *camaldulensis,* 2003*, J. Roux* (CMW-IA:5519 = CMW:12675 – culture; PRU:4566 – dried specimen).

*Ceratocystis pirilliformis*: South Africa: *Mpumalanga:* Nelspruit*,* isolated from *Eucalyptus grandis* × *camaldulensis,* 2004*, N.G. Kamgan* (CMW-IA:4944 = CMW:16513 – culture; PRU:4567 – dried specimen).

#### Nucleotide sequence accession number

The annotated genome sequences for *Ceratocystis pirilliformis* (CMW-IA:5519 and CMW-IA:4944) have been deposited at DDBJ/ENA/GenBank under the accession numbers JAWDJO000000000 and JAVVNR000000000, respectively. This paper describes the first versions of these genome assemblies.

#### Results and discussion

The genome assemblies of *C. pirilliformis* CMW-IA:5519 and CMW-IA:4944 were 26.20 Mb and 26.15 Mb, and had a genome coverage of 240X and 150X, respectively. For CMW-IA:5519, there were initially 23 209 627 raw reads, and after trimming low quality reads, 81% of the reads were retained for genome assembly. For CMW-IA:4944 an initial number of 14 806 790 raw reads were trimmed and 83% retained for the assembly. The genome of CMW-IA:5519 assembled to 567 contigs, all exceeding 1 000 bp in length and with at least 100X coverage (Fig. S1), while that of CMW-IA:4944 assembled into 637 contigs above 1 000 bp with at least 67X coverage (Fig. S1). The assembly of CMW-IA:5519 exhibited an N50 value of 90 938 bp and an N90 value of 50 078 bp, with L50 and L75 values of 89 and 188, respectively. For the CMW-IA:4944 assembly, the N50 value was 78 262 bp, and the N75 value 41 589 bp, with corresponding L50 and L75 values of 103 and 216, respectively. Both *C. pirilliformis* strains had a GC content of 48.1%.

Genome completeness was assessed using BUSCO, with both CMW-IA:5519 and CMW-IA:4944 having completeness scores of 98.9% for the fungal dataset, and 91.8% and 97.7% for the *Sordariomycetes* dataset, respectively. There were 166 repeat families identified in CMW-IA:5519 and 6.11% of the genome was repetitive, while there were 162 repeat families in the assembly of CMW-IA:4944, constituting 6.07% of the genome. A total of 7 012 and 7 003 protein-coding genes were predicted for CMW-IA:5519 and CMW-IA:4944, respectively, and of the predicted genes, 83% and 85% were functionally annotated (File S2). The two assemblies shared 5 608 single-copy orthologs. A total of 183 and 185 CAZymes were identified for CMW-IA:5519 and CMW-IA:4944, respectively. Of the CAZymes identified, the GH16 family, which acts on glucans and galactans, had the highest copy number. In both strains, four mating-type genes, namely *MAT1-1–1*, *MAT1-1–2*, *MAT1-2–1*, and *MAT1-2–7*, were identified on a single contig, suggesting a homothallic reproductive system in this species.

**Fig. 2:**
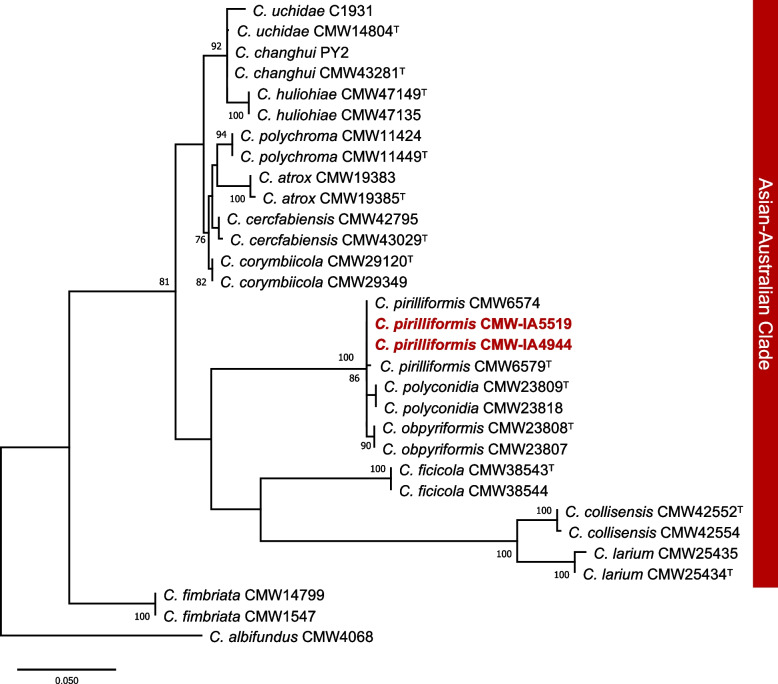
Maximum Likelihood (ML) tree based on the ITS region showing the position of *Ceratocystis* species in the Asian-Australian Clade (AAC). The ITS sequences extracted from the genomes of the two *Ceratocystis pirilliformis* strains from South Africa are highlighted in red bold text and group with other *C. pirilliformis* isolates, including the ex-type strain of the species. Bootstrap support values above 70%, generated from 1000 replications, are indicated at the branches. ^T^ denotes ex-type strains

Phylogenetic analyses confirmed the identity of the two isolates used for genome sequencing. The ITS regions extracted from the two genomes were aligned to those of *C. pirilliformis* from Australia, including the ex-type strain CMW:6579. In the phylogenetic analyses, these sequences formed a distinct subclade with *C. obpyriformis* and *C. polyconidia* within the Asian-Australian Clade (Fig. 2).

The two *C. pirilliformis* genomes sequenced in this study complete the set of genomes made available for strains representing the four phylogenetic clades in *Ceratocystis*. These representative genomes can now be used to investigate various questions via comparative genomics, such as the mechanisms of host specificity and pathogenicity, and the evolutionary history of the different clades. They can also be mined to develop diagnostic tools and population genetic markers

(*Authors*: Irene Barnes*, Anja Piso, Lina Del Mar Angel, and Daniella Krämer*

*Contact: irene.barnes@fabi.up.ac.za; daniella.kramer@fabi.up.ac.za).

## IMA GENOME-F 19B

### Draft genome assemblies of *Diaporthe australafricana* isolates CMW-IA:616 and CMW-IA:644

The genus *Diaporthe* represents a diverse group of important plant pathogens, endophytes, and saprobes (Udayanga et al. 2011; Gomes et al. 2013). These species occur on a wide range of plants and are widely distributed in tropical and temperate regions of the world. Based on multi-locus sequence analyses, a contemporary view recognises 13 species complexes within *Diaporthe* (Norphanphoun et al. 2022). Of these, the stem canker pathogen *Diaporthe australafricana* resides in the *Diaporthe rudis* species complex. This pathogen occurs on blueberry and hazelnut in Chile (Guerrero and Pérez 2013; Elfar et al. 2013) and grapevine in South Africa, Australia, and northern California (Van Niekerk et al. 2005; Gomes et al. 2013; Lawrence et al. 2015).

*Diaporthe* is a diverse paraphyletic group including more than 280 species supported by ex-type cultures and DNA sequence databases (Gao et al. 2017; Bhunjun et al. 2022). Due to its diverse nature, *Diaporthe* species are considered an important source of diverse and bioactive metabolites (Chepkirui and Stadler 2017). The genus thus shows great potential for the discovery of novel secondary metabolites, although the ecological roles of these secondary metabolites remain poorly studied.

Previous research on *Diaporthe* has mostly focused on their taxonomy and there are few species for which whole genomes have been shared on publicly available databases. In this regard, genomic resources offer opportunities to obtain a deeper understanding of plant-pathogen interactions and the dual pathogen-endophyte lifestyles known to occur in *Diaporthe* (Hilário et al. 2023). Genome data will also facilitate increases in understanding of secondary metabolite production and the ecological interactions between *Diaporthe* species and their plant hosts. Currently, genomes are publicly available for 16 *Diaporthe* species (Hilário et al. 2023; Muterko et al. 2023). The aim of this study was to add to this resource by providing the whole genome sequences for two *D. australafricana* strains.

#### Sequenced strains

*Diaporthe australafricana:* South Africa: *Western Cape Province:* Helderberg Nature Reserve, S -34.062488509756164, S 18.874624957427777 isolated from isolated from *Restio quadratus*, 13 April 2002, S. *Lee* (CMW-IA:644 = CMW:18293).

*Diaporthe australafricana:* South Africa: *Western Cape Province:* Helderberg Nature Reserve*,* S -34.062488509756164, S 18.874624957427777 isolated from *Begia capensis*, 13 April 2002, S. *Lee* (CMW-IA:616 = CMW:18300 – culture; PRU:4568 – dried specimen).

#### Nucleotide sequence accession numbers

The annotated genome sequences of *Diaporthe australafricana* (CMW-IA:644 and CMW-IA:616) have been deposited at Genbank under the Accession Numbers JAVXZA000000000 and JAWRVE000000000, respectively (Bioproject PRJNA1005571 and Biosamples SAMN37000851 and SAMN37000852). The versions presented here are the first versions.

#### Results and discussion

The assembled genome size of *D. australafricana* CMW-IA:644 was 51.23 Mb, with an N50 of 264 031 bp and L50 of 62. The assembly consisted of 459 contigs, all of which were above 1 000 bp in length, with a coverage of at least 15X (Fig. S2) and a GC content of 53.25%. An approximate genome coverage of 115X was achieved for CMW-IA:644. A total of 14 374 protein coding genes were predicted of which 11 098 (76.39%) proteins were functionally annotated (File S2). The BUSCO analysis showed that the assembly was 98.8% and 97.5% complete with respect to the fungi and *Sordariomycetes* datasets.

The assembled genome size of *D. australafricana* CMW-IA:616 was 50.80 Mb, with an N50 of 251 366 bp and L50 of 64. The assembly consisted of 505 contigs above 1 000 bp, with a coverage of at least 15X (Fig. S2) and a GC content of 53.37%. An approximate genome coverage of 135X was achieved. A total of 14 404 protein coding genes were predicted, of which 10 991 (76.31%) were functionally annotated (File S2). The BUSCO analysis showed that the assembly was 98.3% and 97.6% complete with respect to the fungi and *Sordariomycetes* datasets.

**Fig. 3:**
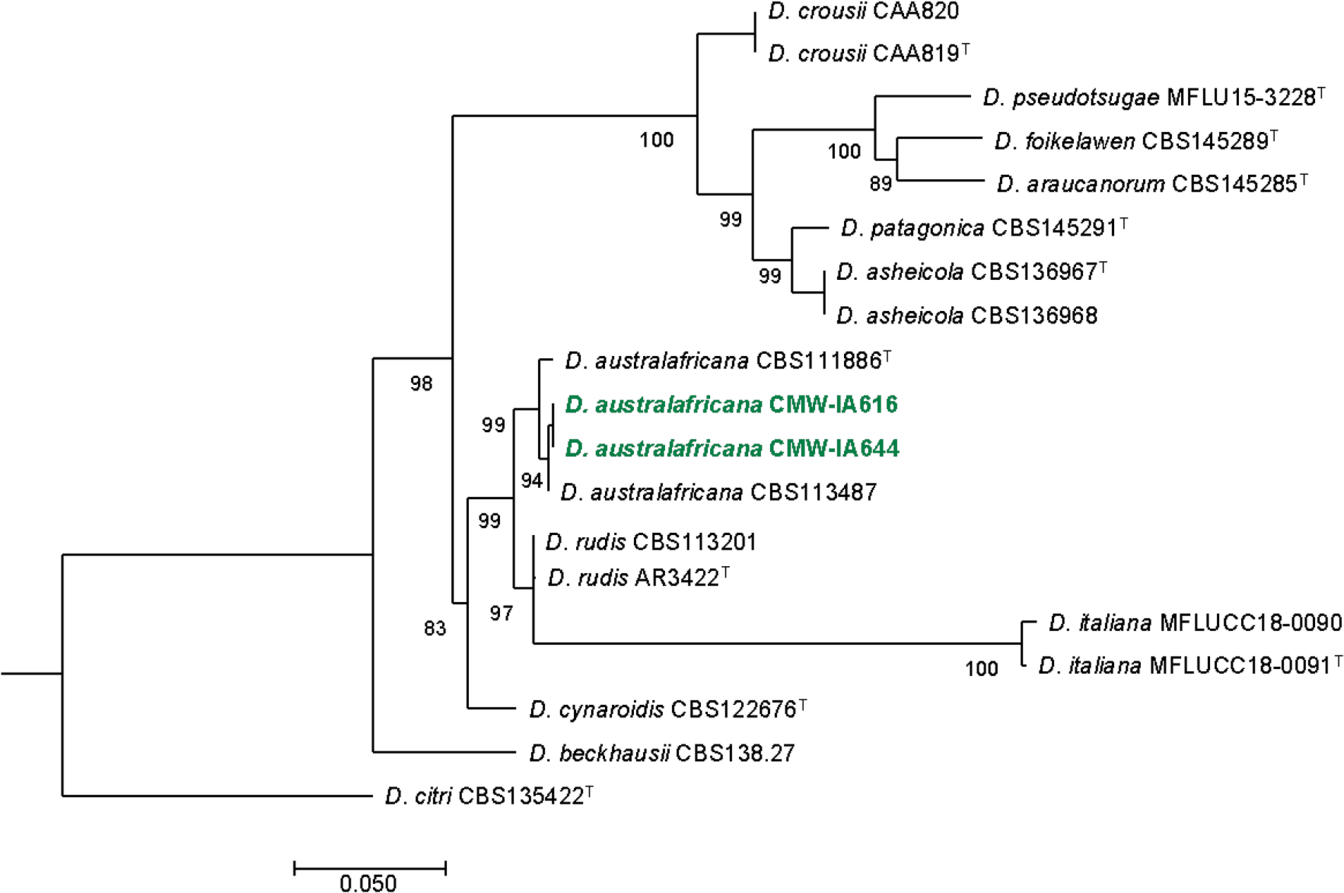
Maximum Likelihood tree based on the partial gene sequences of β-tubulin, calmodulin and translation elongation factor 1-α. Values at branch nodes are the bootstrapping confidence values, showing those ≥ 80%. The *Diaporthe australafricana* isolates sequenced in this study are indicated in green, bold text. ^T^ indicates ex-type strains

Genome comparisons between the two isolates indicated 9 779 shared single-copy orthologs. A total of 783 and 789 CAZymes were identified in the CMW-IA:644 and CMW-IA:616 assemblies, respectively (File S2). The most abundant CAZyme families were AA7 and AA3, which is typical for wood-degrading fungi. ML analysis of two concatenated barcoding gene regions confirmed the identity of CMW-IA:644 and CMW-IA:616 as *D. australafricana* (Fig. 3). Genes from both the *MAT1-1* and *MAT1-2* idiomorphs were on Scaffold_206 of CMW-IA:644 and Scaffold_105 of CMW-IA:616, suggesting that *D*. *australafricana* has a homothallic reproductive system. Species identification, phylogenetic reconstruction, and the subsequent establishment of species limits within *Diaporthe* are dependent on multi-locus sequence data (Gomes et al. 2013; Gao et al. 2017; Norphanphoun et al. 2022). Not all informative regions have been sequenced for *Diaporthe* species and genome data can help to overcome this limitation

(*Authors*: Byron Sonnekus*, WenWen Li, Carla Buitendag, Janneke Aylward, Brenda D. Wingfield, and Michael J. Wingfield

 *Contact: Byron.sonnekus@fabi.up.ac.za).

## IMA GENOME-F 19C

### Draft genome assemblies of *Fusarium ophioides* isolates CMW-IA:4746, CMW-IA:5006, and CMW-IA:5007.

Species within the *Fusarium fujikuroi* species complex (FFSC) are important pathogens of agricultural and forestry crops (Herron et al. 2015; Yilmaz et al. 2021; Han et al. 2023). Some members of this complex can produce mycotoxins that are secondary metabolites harmful to humans and animals (Munkvold et al. 2021; Yilmaz et al. 2021). One of the species belonging to the FFSC, *Fusarium ophioides*, was discovered during a comprehensive survey of fungal diversity on *Panicum maximum*, a native grass growing near a pine seedling nursery in South Africa. This discovery followed a significant outbreak of Pitch canker caused by *Fusarium circinatum* (Jacobs 2010). The original taxonomic description of *F. ophioides* by Jacobs (2010) was invalid because it failed to include an ISSN number, which is a requirement for legitimate publication under Article 30.9 of the International Code of Nomenclature for algae, fungi, and plants (Turland et al. 2018). Yilmaz et al. (2021) subsequently validated the taxonomic status of this species.

Phylogenetically, *F. ophioides* resides in the American Clade (Yilmaz et al. 2021) and is genetically closely related to pathogenic species that affect mango and pineapple, such as *F. mexicanum*, *F. sterilihyphosum* and *F. ananatum* (Britz et al. 2002; Jacobs et al. 2010; Otero-Colina et al. 2010). Other than morphology and ecology described in the previous studies, knowledge regarding *F. ophioides* remains limited. Therefore, sequencing the genome of this species is relevant as it provides the basis for an in-depth exploration of its genetic composition, broader biological traits, and evolutionary history. The aim of this study was to sequence the complete genomes of three *F. ophioides* isolates, providing the basis for future studies.

#### Sequenced strains

*Fusarium ophioides:* South Africa: *Ngodwana*: isolated from *Panicum maximum*, 1 Nov 1994, *G.H.J. Kemp* (CMW-IA:5007 = CMW:18679 = CBS:118510 — culture; PRU:4569 – dried specimen; CMW-IA:5006 = CMW:18681 = CBS:118512 — ex-type strain; PRU:4570 – dried specimen; and CMW-IA:4746 = CMW:18682 — culture; PRU:4571 – dried specimen).

#### Nucleotide sequence accession numbers

The annotated genome sequences of *F. ophioides* have been deposited at GenBank under the accessions SAMN37000853 (CMW-IA:5007), SAMN37000854 (CMW-IA:5006) and SAMN37000855 (CMW-IA:4746). This paper describes the first versions.

#### Results and discussion

**Fig. 4:**
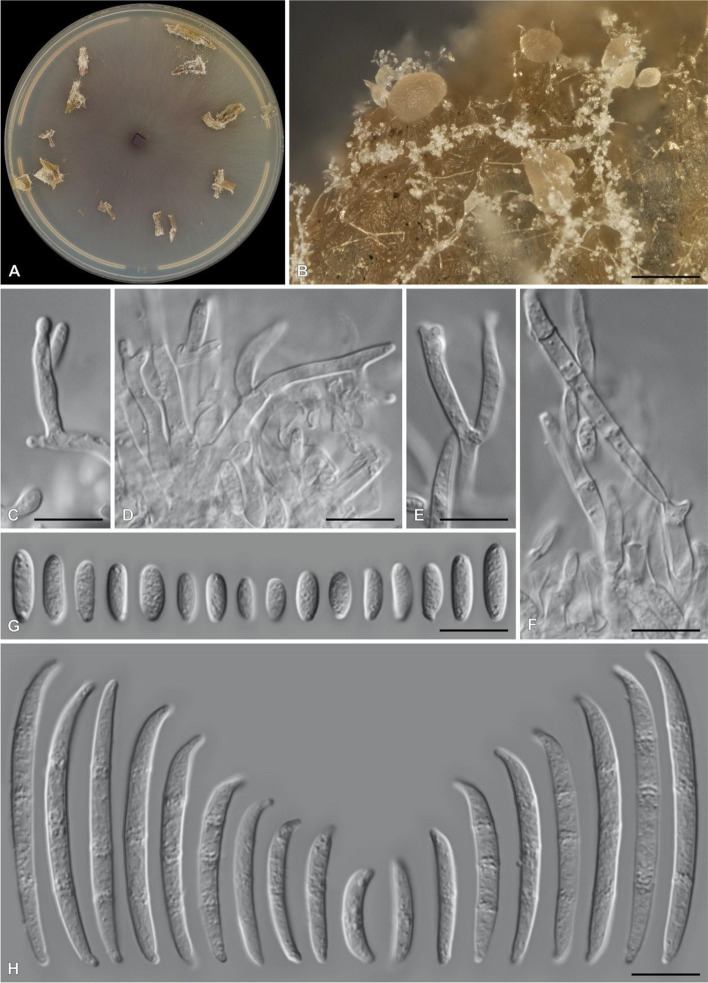
Photoplate of *Fusarium ophioides* (CMW-IA:5006). **A**. Colony grown for 7 days at 25 °C in intermittent near-UV-light on water agar with carnation leaves. **B**. Sporodochia and aerial mycelia produced on carnation leaves. **C**–**F**. Conidiophores with mono- and polyphialides. **G**. Aerial microconidia. **H**. Sporodochial macroconidia. Bars: B = 100 µm; **C**–**H** = 10 µm

The sequenced *F. ophioides* strains displayed morphological characters typical of the genus (Fig. 4). The genome assembly statistics of the three isolates are summarized in Table 1. Contigs of at least 1 000 bp and with at least 25X coverage were retained for further analysis (Fig. S3). The genome assemblies of the three *F. ophioides* isolates from South Africa were 43.5 Mb with approximately 50X coverage, 44.3 Mb with 115X coverage and 44.52 MB with 15X coverage for CMW-IA:4746, CMW-IA:5006 and CMW-IA:5007, respectively. The N50 and L50 was in the same range for all three genomes (Table 1). For all three genomes, BlobToolKit did not detect significant contamination (Fig. S3) and the estimated completeness of the three genomes varied from 97.2% at the lowest taxonomic level (*Hypocreales*) to 98.2% at the highest level (Fungi). Gene prediction and functional annotation statistics for genomes are listed in Table 1.

**Table 1:** A summary of the genome statistics for the three *Fusarium ophioides* genomes presented in this study

		**CMW-IA:4746**	**CMW-IA:5006**	**CMW-IA:5007**
**Genome assembly**	Assembly size (Mb)	43.5	44.3	44.5
	Number of contigs	185	259	323
	N50 (kb)	432 242	379 241	417 843
	L50	30	36	33
	Estimated Coverage	50X	115X	15X
	GC content (%)	48.8	48.7	48.7
**Genome completeness**	Fungi odb10 (%)	98.2	98.5	98.8
	*Hypocreales* odb10 (%)	97.2	97.7	97.8
**Gene Prediction**	Protein-coding	14 361	15 044	15 047
	tRNA genes	302	304	305
**Functional annotation**	Number of proteins with Enzyme Commission (EC) numbers	2 191	2 237	2 250
	Number of proteins with InterPro (IPR) domains	10 804	11 188	11 182

Comparisons among the three *F. ophioides* strains revealed that they shared 9 520 single-copy orthologs. For CMW-IA:4746, CMW-IA:5006 and CMW-IA:5007, the percentage of predicted proteins with either an IPR domain or EC number annotation was 90.49%, 89.24% and 89.27%, respectively (File S2). For all three genome assemblies, CAZyme family AA7, a family of gluco-oligosaccharide oxidases, was by far the most abundant. CMW-IA:4746 had a smaller genome assembly, less predicted genes and less CAZymes and proteases than the other two assemblies. This may be due to the lower completeness of the genome as measured by BUSCO analysis (Table 1).

**Fig. 5:**
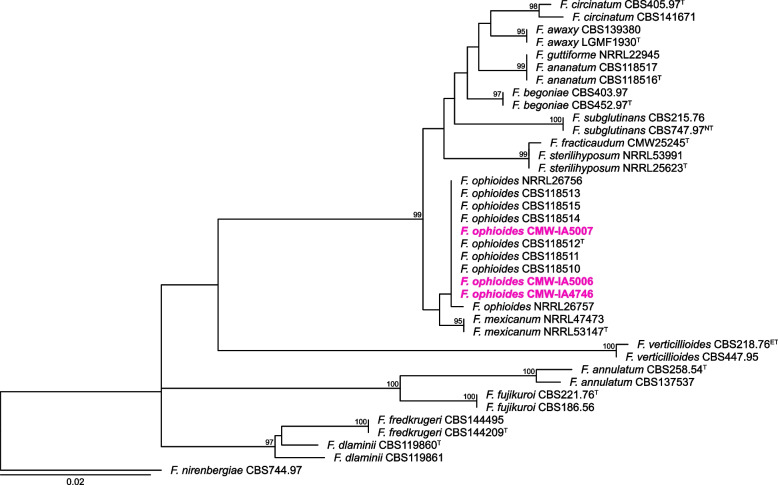
Phylogenetic tree of selected *Fusarium fujikuroi* species complex based on *TEF1*. The tree was rooted to *F. nirenbergiae* (CBS:744.97). The *F. ophioides* strains sequenced in this study are shown in pink coloured bold text. Branch support in nodes higher than 80% bs are indicated above relevant branches. ^T^ indicates ex-type, ^NT^ indicates neotype, and ^ET^ indicates epitype strains

A phylogenetic analysis of the *TEF1* gene region confirmed that the three strains sequenced belong to *F. ophioides*, resolve in the “American Clade” and are sister to *F. mexicanum* (Fig. 5). All three isolates harboured genes from the *MAT1-1* mating-type locus, suggesting that this species is heterothallic. The genomes of *F. ophioides* generated in this study will add to the already growing genome resources for the genus *Fusarium*. This will play an important role in resolving questions regarding the taxonomy of the genus and provides a better understanding of their shared evolutionary history, biology, and ecology (*Authors*: Neriman Yilmaz*, Alida van Dijk, Jenna-Lee Price, Kiara Munsamy, Katharina Gasser, and Cobus M. Visagie

*Contact: neriman.yilmazvisagie@fabi.up.ac.za).

## IMA GENOME-F 19D

### Draft genome assemblies of *Paecilomyces lecythidis* isolates CMW-IA:5739 and CMW-IA:550

*Paecilomyces* [MB#9196] was described for fungi with branched conidiophores producing divergent whorls of phialides that can either be cylindrical or have inflated bases that taper to long and distinct necks (commonly bent away from the phialide axis) and producing pale yellow–brown conidia. Brown & Smith (1957) monographed the genus and included the insect-pathogenic *Isaria farinosa* [MB#156563], which was followed by Samson (1974) accepting 31 species in his subsequent revision of the genus. *Byssochlamys* [MB#702] was widely considered as the sexual state of *Paecilomyces* (Brown & Smith 1957; Stolk & Samson 1971; Samson 1974). *Paecilomyces* was later shown to be polyphyletic based on an 18S rDNA phylogeny, with *P. variotii* [MB#248517] (generic type) belonging in *Eurotiales*, *P. farinosus* (≡ *Isaria farinosa*) [MB#302185] in *Hypocreales*, *P. inflatus* [MB#335528] in *Sordariales* (Luangsa-Ard et al. 2004), and later *P. lilacinus* (as *Purpureocillium*) in *Hypocreales* [MB#519529] (Luangsa-Ard et al. 2011).

After the "One Fungus = One Name" concept was adopted in the International Code of Nomenclature for algae, fungi, and plants (Hawksworth et al. 2011; McNeill & Turland 2011), a decision was made to adopt the older name *Paecilomyces* over *Byssochlamys* (Houbraken et al. 2020). At the time, Houbraken et al. (2020) accepted 10 species, including *P. brunneolus* [MB#512559], *P. dactylethromorphus* [MB#302183], *P. divaricatus* [MB#512561], *P. fulvus* [MB#319107], *P. lagunculariae* [MB#832559], *P. niveus* [MB#319117], *P. paravariotii* [MB#846976], *P. tabacinus* [MB#816870], *P. variotii* and *P. zollerniae* [MB#319129]. *Paecilomyces clematidis* [MB#843540], *P. formosus* [MB#846977] and *P. penicilliformis* [MB#834874] were described more recently (Crous et al. 2020; Spetik et al. 2022; Urquhart & Idnurm 2023). Heidarian et al. (2018) and Houbraken et al. (2020) suggested that *P. formosus* could represent three species. This was confirmed by whole genome comparisons for representative strains in the clade and resulted in the re-introduction of two old names *P. lecythidis* [MB#335530] and *P. maximus* [MB#335531], and a validated *P. formosus* [MB#846977] (Urquhart & Idnurm 2023).

*Paecilomyces* is classified in *Thermoascaceae* (*Eurotiales*) and can be found across the world from various indoor and environmental sources. Species are typically thermophilic and/or xerophilic, a character(s) that makes them effective food spoilers of heat processed, acidic foods (Pitt & Hocking 2009; Houbraken & Samson 2011). Apart from foods, species also grow on and damage items made of wood, leather, paper and textiles (Brown & Smith 1957). Similarly, *P. formosus *sensu lato (Samson et al. 2009) can be found on a wide range of substrates and has occasionally been reported to cause disease in humans (Heshmatnia et al. 2017), animals (Anderson et al. 2022) and trees (Heidarian et al. 2018; Sabernasab et al. 2019; Rostami & Jamali 2023), and it has also been shown to have potential as a plant growth promotor (Khan et al. 2012). Here we report two genomes of *P. lecythidis*.

#### Sequenced strains

*Paecilomyces lecythidis:* South Africa: *Stellenbosch*: isolated from a wooden utility pole, 16 Oct 2013, E. de Meyer (CMW-IA:550 = CMW:18167 = KF22SB1 = CN175G7 — culture; PRU:4575 — dried specimen).

*Paecilomyces lecythidis:* South Africa: *Kimberley*: isolated from a wooden utility pole, 17 Sept 2013, E. de Meyer (CMW-IA:5739 = CMW:18170 = K15EB1 = CN175G9 — culture; PRU:4574 — dried specimen).

#### Nucleotide sequence accession numbers

The annotated genome sequences of *P. lecythidis* have been deposited at GenBank under the accessions SAMN36999128 (CMW-IA:550) and SAMN36999129 (CMW-IA:5739). This paper describes the first versions.

#### Results and discussion

**Fig. 6:**
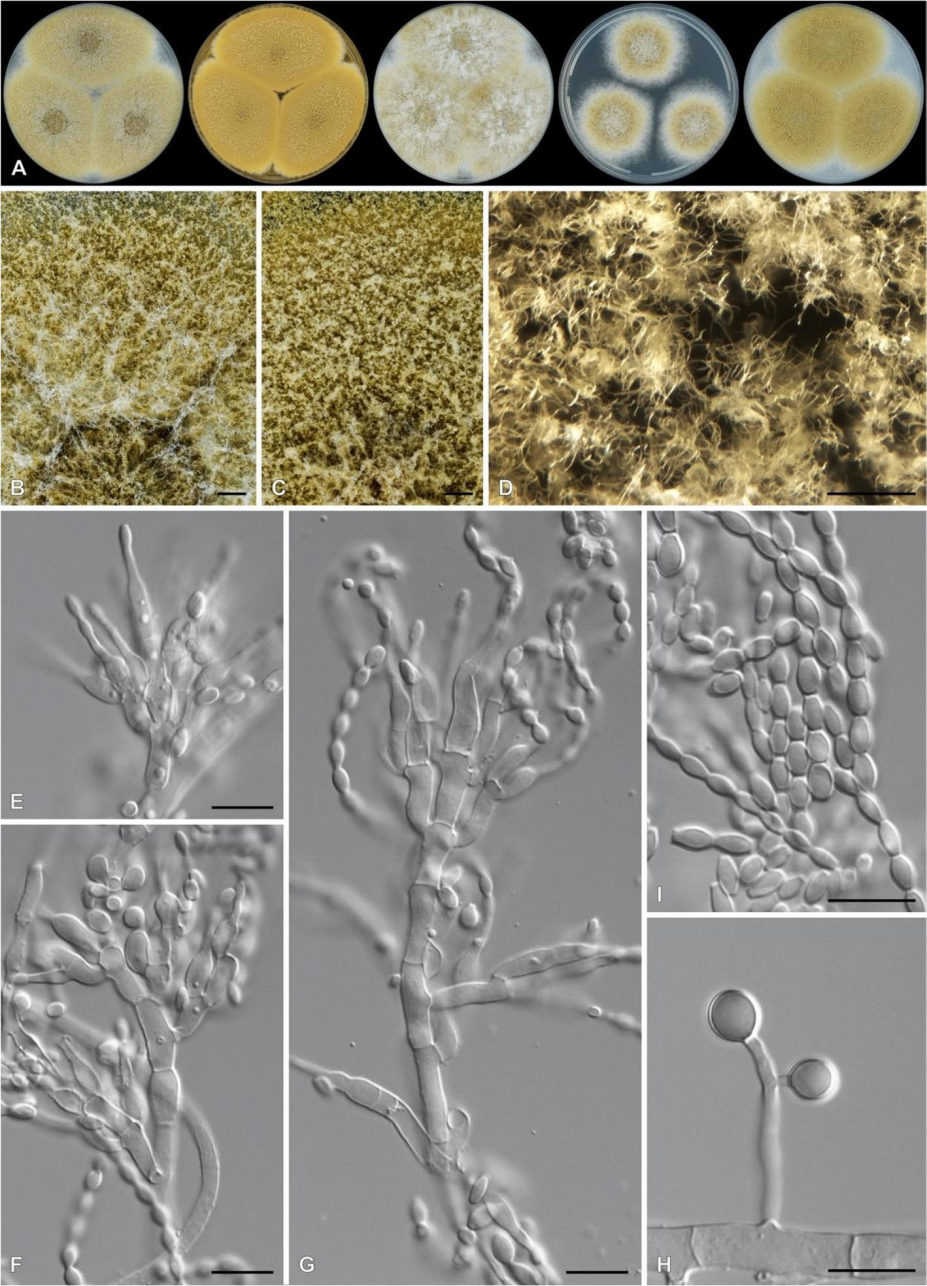
Photoplate of *Paecilomyces lecythidis* (CMW-IA:550). **A**. Colonies (from left to right) grown on Czapek yeast autolysate agar, malt extract agar, yeast extract sucrose agar, dichloran 18% glycerol agar and oatmeal agar, for 7 days at 25 °C in darkness. **B**. Close-up of CYA colony. **C**, **D**. Close-ups of MEA colonies. **E**–**G**. Conidiophores. **H**. Chlamydospores. **I**. Conidia. Bars: **B**, **C** = 1 mm; **D** = 250 µm; **E**–**I** = 10 µm

The sequenced *P. lecythidis* strains displayed morphological characters typical of the genus (Fig. 6). Sequencing of CMW-IA:550 yielded 12 754 825 reads with a length of 2 × 151 bp and FastQC did not flag any low-quality or overrepresented sequences. The final 30.96 Mb assembly had a GC content of 48.74%, a coverage of approximately 85X, comprising 98 contigs above 1 000 bp, with an L50 of 14 and an N50 of 674 067 bp. Contigs with less than 15X coverage were filtered from the genome using BlobToolKit (Fig. S4). Genome completeness according to the fungi_odb10 dataset was estimated at 98.2% corresponding to 97.4% complete and single-copy BUSCOs, 0.8% complete and duplicated BUSCOs, 0.7% fragmented BUSCOs and 1.1% missing BUSCOs. Genome completeness according to the eurotiales_odb10 dataset was estimated at 94.8% corresponding to 94.3% complete and single-copy BUSCOs, 0.5% complete and duplicated BUSCOs, 0.6% fragmented BUSCOs and 4.6% missing BUSCOs. RepeatModeler identified 1.46% of the genome as repetitive and Funannotate predicted 9 735 protein-coding and 157 tRNA genes.

Sequencing of CMW-IA:5739 yielded 11 817 854 million reads with a length of 2 × 151 bp and FastQC did not flag any low-quality or overrepresented sequences. The final 31.27 Mb assembly had a GC content of 48.55%, a coverage of approximately 85X, comprising 88 contigs above 1 000 bp, with an L50 of 14 and an N50 of 719 368 bp. Contigs with less than 15X coverage were filtered from the genome using BlobToolKit (Fig. S4). Genome completeness according to the fungi_odb10 dataset was estimated at 98.4% corresponding to 97.8% complete and single-copy BUSCOs, 0.9% complete and duplicated BUSCOs, 0.5% fragmented BUSCOs and 0.8% missing BUSCOs. Genome completeness according to the eurotiales_odb10 dataset was estimated at 96.8% corresponding to 96.5% complete and single-copy BUSCOs, 0.3% complete and duplicated BUSCOs, 0.6% fragmented BUSCOs and 2.6% missing BUSCOs. RepeatModeler identified 1.91% of the genome as repetitive and Funannotate predicted 9 646 protein-coding and 165 tRNA genes.

A total of 8 102 out of 9 578 (84.59%) predicted proteins were functionally annotated using Funannotate for CMW-IA:550, while CMW-IA:5739 had 8 218 of 9 646 (85.20%) functionally annotated proteins. The two strains shared 6 572 single-copy orthologs. The number of protease families was similar between both genomes, but the Auxiliary Activity CAZyme families AA1, AA3 and AA7 had a much higher frequency in the CMW-IA:5739 assembly. The main function of these families in fungi is to assist in the degradation of complex carbohydrates, such as cellulose and chitin (Levasseur et al. 2013). This same trend was seen with the Glycoside hydrolase families, with GH3, GH13, GH18, and GH43 having notably higher frequencies in CMW-IA:5739 (File S2).

**Fig. 7:**
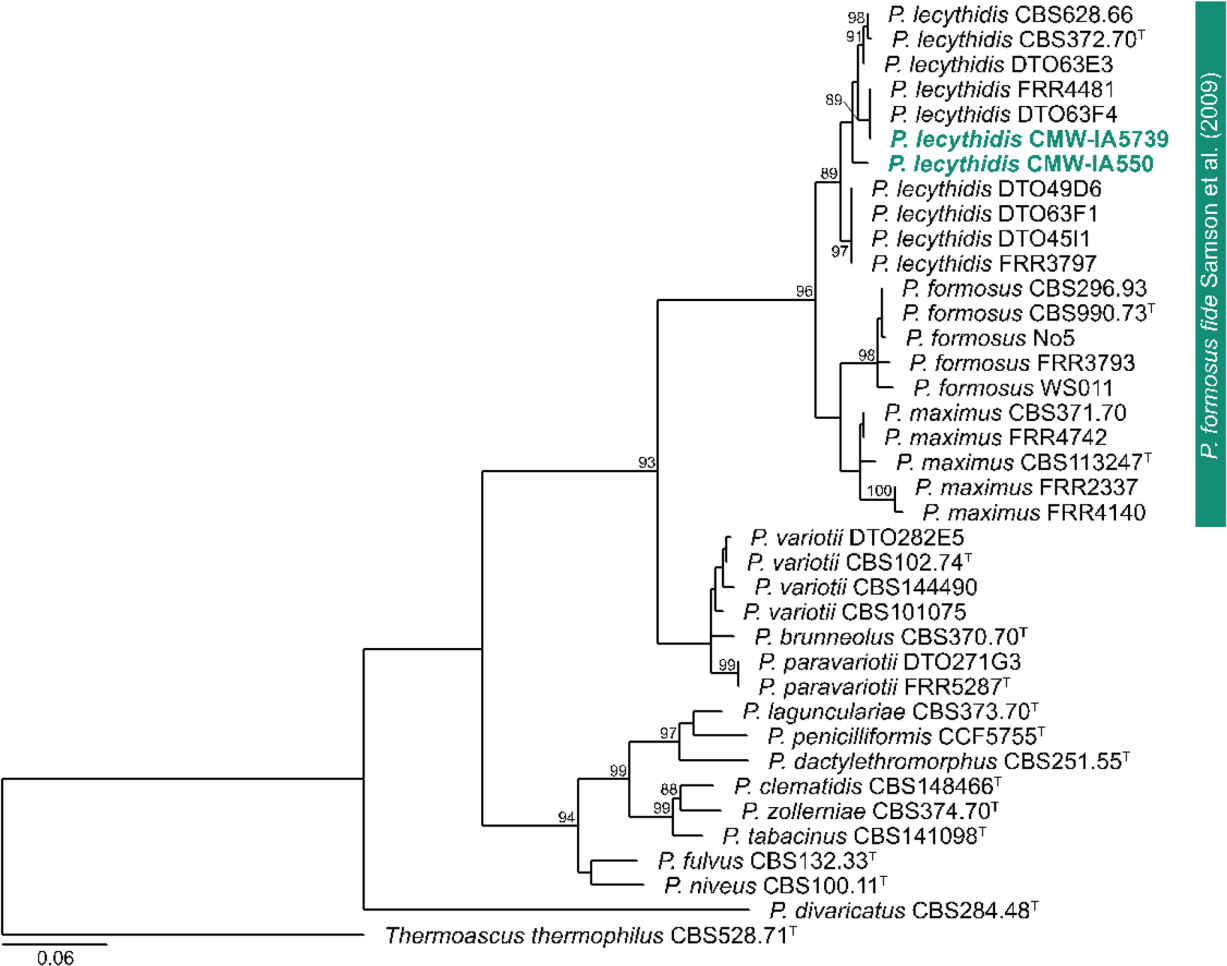
Phylogenetic tree of *Paecilomyces* based on *BenA*. The tree was rooted to *Thermoascus thermophilus*. The *Paecilomyces lecythidis* strains that were sequenced here are shown in coloured bold text. Branch support in nodes higher than 80% bs are indicated above relevant branches. ^T^ indicates ex-type strains

A phylogenetic analysis of the *BenA* gene region confirmed that strains resolve in three main clades that Urquhart & Idnurm (2023) named *P. formosus, P. lecythidis* and *P. maximus*. The strains sequenced in this study belonged to *P. lecythidis* (Fig. 7). The genome sequences of *P. lecythidis* brings the total number of genomes for the species to four and for the genus to 48 (Urquhart & Idnurm 2023). These cover eight of the 15 accepted species, including *P. dactylethromorphus* (*n* = 3), *P. formosus* (*n* = 3), *P. fulvus* (*n* = 1), *P. lecythidis* (*n* = 4), *P. maximus* (*n* = 3), *P. niveus* (*n* = 1), *P. paravariotii* (*n* = 2), and *P. variotii* (*n* = 31). *P. lecythidis* appears to be heterothallic, with both strains harbouring the *MAT1-1* idiomorph. These *Paecilomyces* genomes will play an important role in future studies aimed at elucidating the taxonomy of the genus and understanding their biology more completely

(*Authors*: Cobus M. Visagie*, Nicole van Vuuren, and Callin Ceriani

*Contact: cobus.visagie@fabi.up.ac.za).

## IMA GENOME-F 19E

### Draft genome assemblies of *Sporothrix stenoceras* isolates CMW-IA:5313 and CMW-IA:5364

The genus *Sporothrix* includes at least 67 species (De Beer et al. 2022; Bilański et al. 2023). The majority of these reside in clades from plant or soil, but the genus also includes dimorphic agents of human and animal disease (Hektoen & Perkins 1900; Rodrigues et al. 2020). Multigene sequencing has allowed the taxonomy of *Sporothrix* to be significantly better understood, with the identification of several clades and descriptions of many cryptic species in the genus. Within these clades, the major mammal pathogens separate phylogenetically from those known only as saprotrophs (De Beer et al. 2016).

*Sporothrix* has received substantial attention in the last decade, due to the current sporotrichosis epidemics in South America and Asia. In South America, the epidemic is being driven by feline-to-human transmission (De Lima Barros et al. 2004; Rabello et al. 2022), while in Asia, sapronotic transmission of the pathogens persists (Moussa et al. 2017; Yao et al. 2020). The increased incidence of zoonotic transmission as well as the development of antifungal resistance is of concern, and this may contribute to more serious outbreaks in endemic areas in the future (Flórez-Muñoz et al. 2019; Rodrigues et al. 2020; Waller et al. 2021; Teixeira et al. 2022; Bombassaro et al. 2023).

Comparative genomics approaches have attempted to uncover the genetic factors that drive the success of the pathogenic clade in a mammal host. These comparisons have identified genes potentially involved in pathogenicity, thermo-tolerance, and evasion of the host immune system (Barros et al. 2014; Teixeira et al. 2014; Huang et al. 2020; Prakash et al. 2020). Furthermore, these have contributed to the current knowledge on the ecology and evolutionary history of the mammal pathogens in the genus. The three draft *S. stenoceras* genomes presented here add to the 31 *Sporothrix* genomes that are already publicly available (https://www.ncbi.nlm.nih.gov/assembly/?term=Sporothrix). As additional full genome sequences of *Sporothrix* species become available, more comprehensive genome comparisons will be possible, leading to a better understanding of the etiological agents and the identification of novel therapeutic targets.

#### Sequenced strains

*Sporothrix stenoceras*: South Africa: *Western Cape Province*: Canker on *Malus domestica*, 1999, *A. Smith* (CMW-IA:5313 = CMW:5344 – culture; PRU:4572 – dried specimen; CMW-IA:5347 = CMW:5346 – culture; PRU:4573 – dried specimen; and CMW-IA:5364 = CMW:5347 – culture).

#### Nucleotide sequence accession numbers

The annotated genome sequences for *Sporothrix stenoceras* (CMW-IA:5313, CMW-IA:5347 and CMW-IA:5364) have been deposited at DDBJ/ENA/GenBank under the following accession numbers: JAWCWV000000000 (CMW-IA:5313), JAWCUI000000000 (CMW-IA:5347), and JAWCTW000000000 (CMW-IA:5364). This paper describes the first versions.

#### Results and discussion

The length of the *Sporothrix stenoceras* genome assembly for isolate CMW-IA:5313 was 39.5 Mb, CMW-IA:5347 was 39.2 Mb and CMW-IA:5364 was 39.5 Mb. These were assembled into 155, 165 and 139 scaffolds, respectively, all exceeding 1 000 bp and with at least 50X coverage (Fig. S5). Isolate CMW-IA:5313 had an N50 of 447 009 bp and L50 of 27. CMW-IA:5347 had an N50 451 359 bp and L50 of 26, while isolate CMW-IA:5364 had an N50 of 563 416 bp and L50 of 22. All three of the strains had a similar GC content, ranging from 52.8%-52.9%. Based on the sordariomycete_odb10 database, genome completeness of the three isolates was estimated to be 96.5% (CMW-IA:5313) and 96.4% (CMW-IA:5347 and CMW-IA:5364).

A total of 10 400 protein-coding genes were predicted for isolate CMW-IA:5313, at an average density of 263 genes/Mb. Isolate CMW-IA:5347 had 10 258 predicted protein-coding genes, at 261 genes/Mb, and isolate CMW-IA:5364 had 10 397 predictions at an average of 263 genes/Mb. For the respective isolates, 8 626 (82.9%), 8 547 (83.3%) and 8 638 (83.0%) of the predicted genes were functionally annotated. A comparison of the three annotated genomes identified 7 569 shared single-copy orthologs. Analysis of the carbohydrate active enzymes (CAZymes) identified Glycoside Hydrolase Family 3 (GH3) as the most abundant in all three genomes (File S2). This family is known to have a variety of functions, including members with β-D-glucosidase, β-D-xylopyranosidase, α-L-arabinofuranosidase, and N-acetyl-β-D-glucosaminide phosphorylase activities, making the family important in the degradation of oligosaccharides (Harvey et al. 2000; Macdonald et al. 2015; Reichart et al. 2021).

**Fig. 8:**
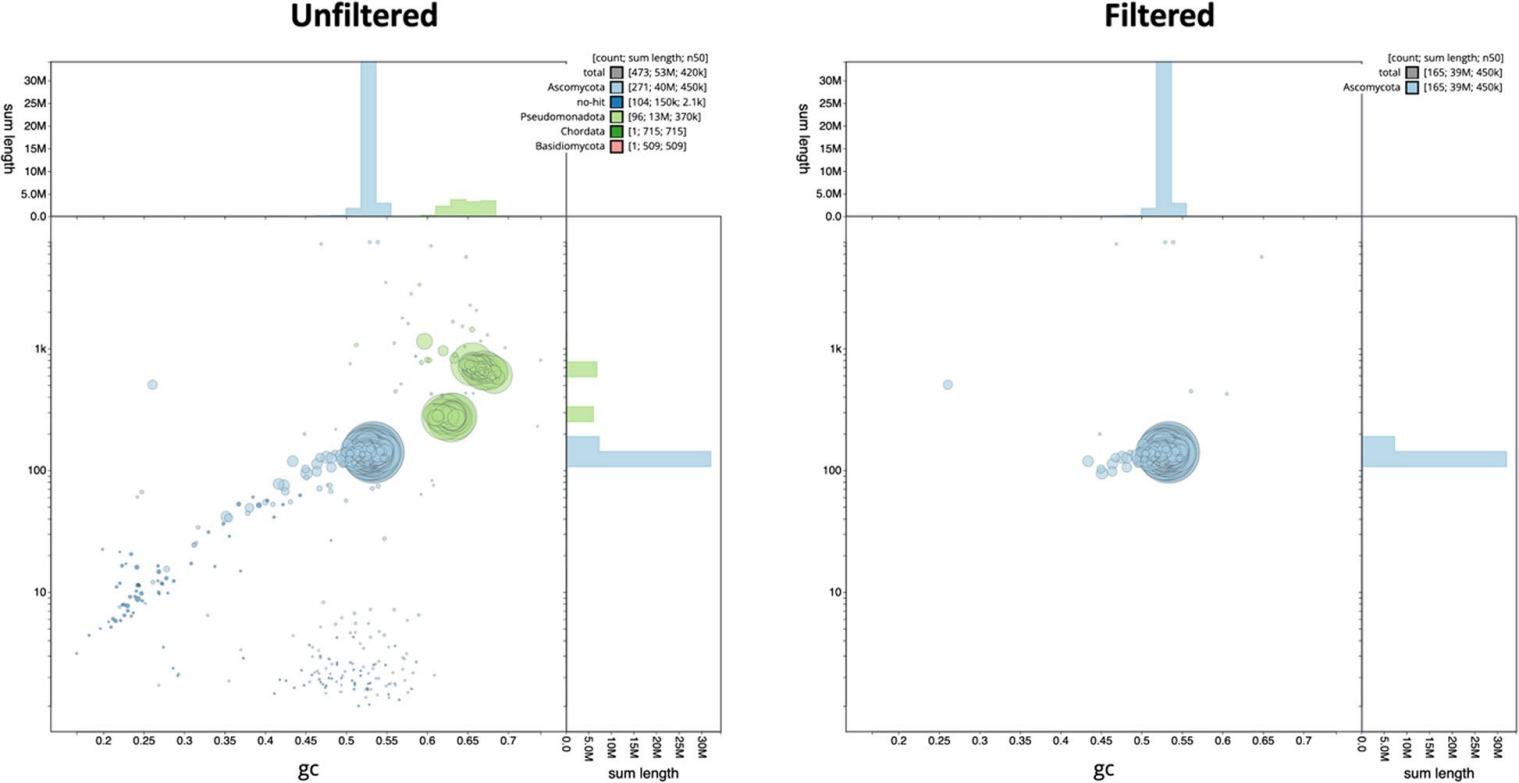
The taxon-annotated GC-coverage plots (BlobPlots) for the *Sporothrix stenoceras* CMW-IA:5347 genome. *Left*: The unfiltered genome, including two clusters (“blobs”) of contigs annotated as “Psuedomonadota” (indicated in green) representing bacterial contamination of this fungal genome. *Right*: The filtered genome, with contigs of < 1 000 bp, with < 50X coverage and those annotated as bacterial in origin removed

Assembly v1.1 of isolate CMW-IA:5347 contained contigs that were annotated as “Psuedomonadota”, indicating that they were of bacterial origin and thus suggesting that the original *S. stenoceras* strain was contaminated with bacterial cells (Fig. 8). It was possible to remove these contigs from the initial assembly using BTK, resulting in a clean assembly with statistics comparable to the other two *S. stenoceras* genomes presented here. This illustrates the power of BTK in the genome assembly pipeline and may prove particularly useful for the assembly of fungi with close bacterial associates (Robinson et al 2021).

**Fig. 9:**
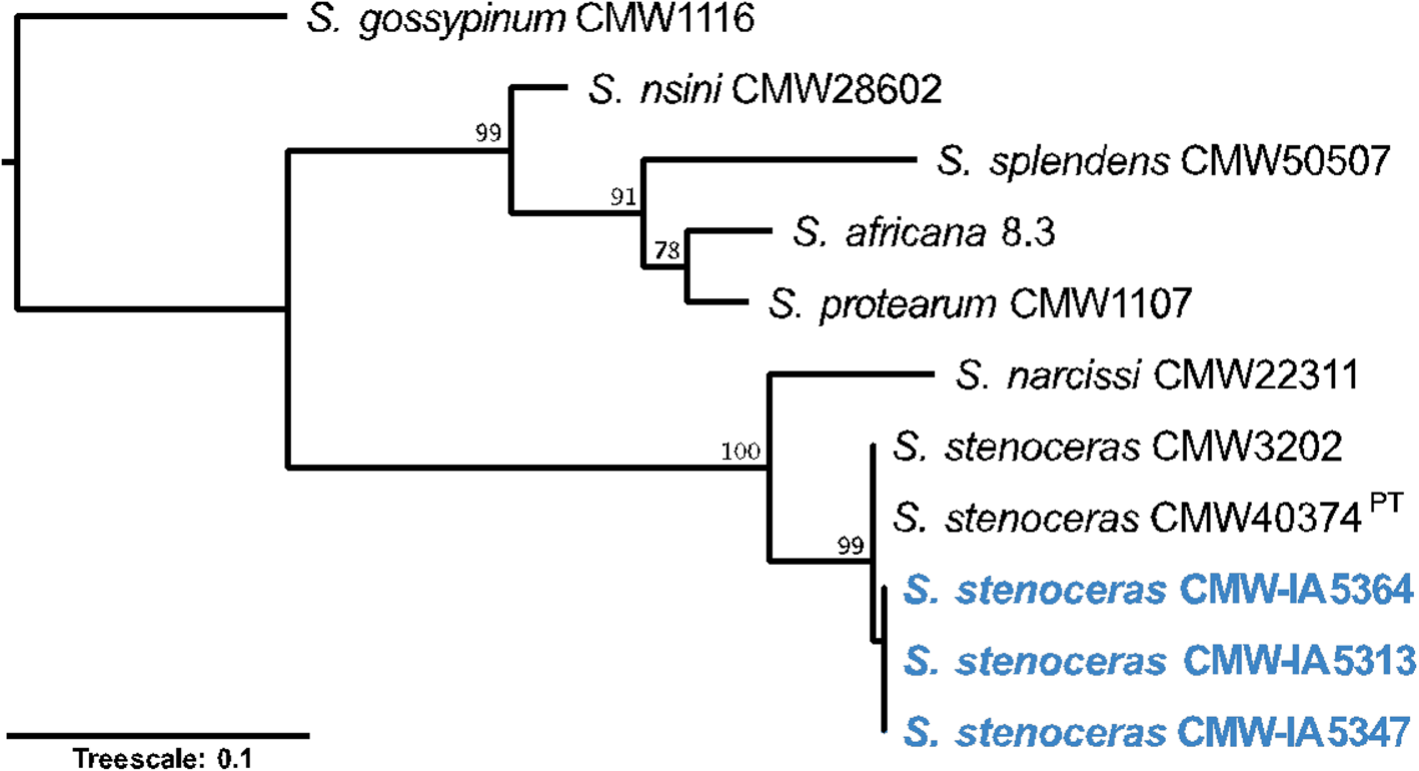
Maximum likelihood tree based on the calmodulin region. The isolates sequenced in this study are indicated in blue and bold. Values on the nodes represent bootstrap confidence values, omitting values < 75%. ^PT^ indicates the ex-paratype isolate

**Fig. 10:**
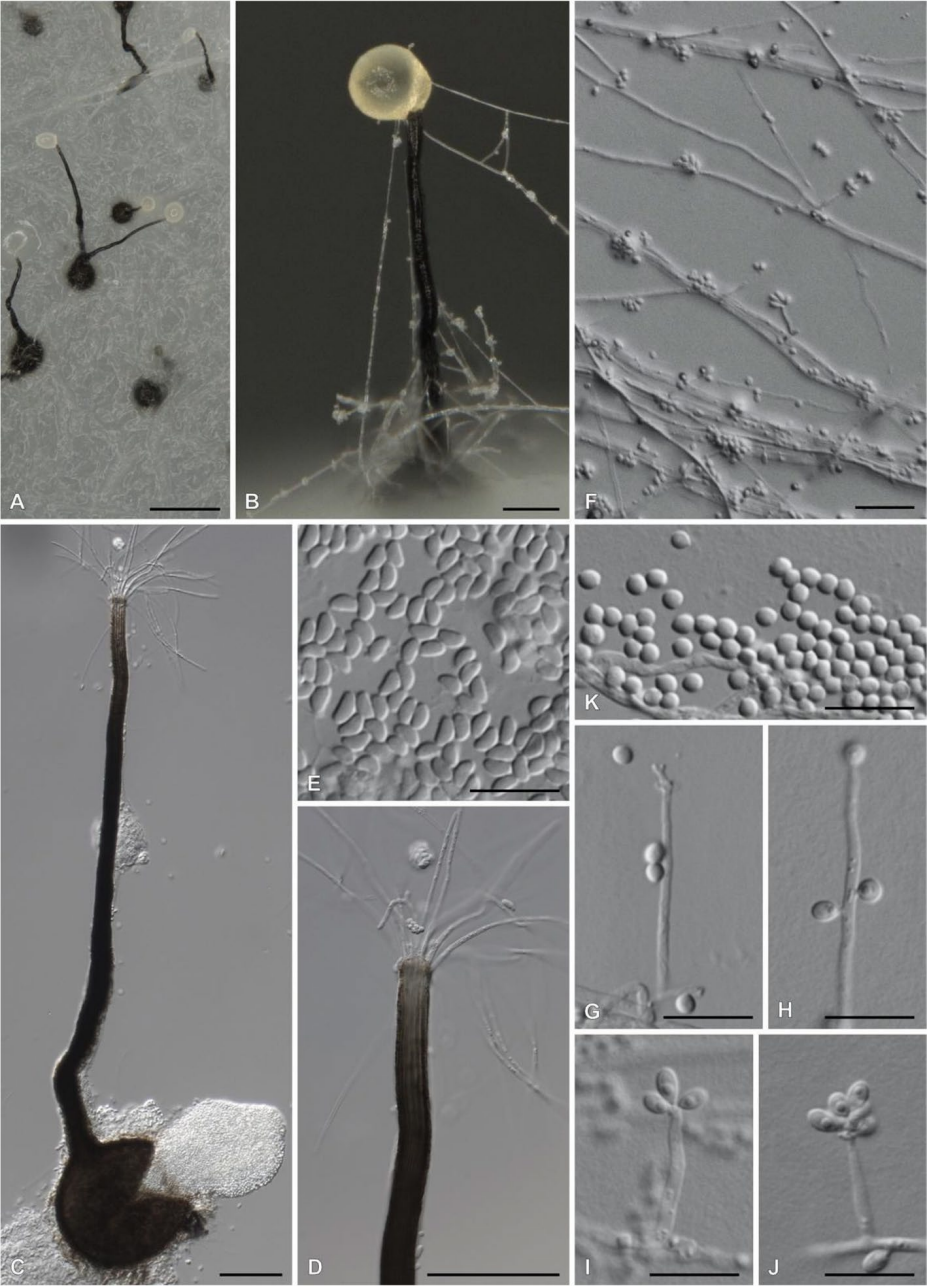
Photoplate of *Sporothrix stenoceras* (CMW-IA:5313). **A**. Close-up of colony grown on PDA for 3 weeks at 25 °C in intermitted light. **B**, **C**. Perithecial ascomata. **D**. Perithicia neck. **E**. Ascospores. **F**–**J**. Conidiophores. **K**. Conidia. Bars: **A** = 250 µm; **B** = 50 µm; **F** = 25 µm; **C**–**E**, **G**–**K** = 10 µm

Phylogenetic analysis confirmed all three isolates to be *S. stenoceras* (Fig. 9). The species has regularly been observed to produce sexual structures (Fig. 10) from pure cultures and has, therefore, been suggested to be self-fertile in nature. All three assemblies were found to harbour genes from both the *MAT1-1* and *MAT1-2* idiomorphs, confirming that the species is homothallic. *S. stenoceras* is thus far the only known *Sporothrix* species to make use of this reproductive strategy. The sexual structures produced by *S. stenoceras* and the historical importance of perithecium and ascus morphology as taxonomic characters, initially led to *S. stenonceras* being classified as a member of the genus *Ophiostoma*, and to the suggestion that it represented the sexual state of the human pathogen *S. schenckii* (Mariat & De Bievre 1968; Andrieu et al. 1971). The inclusion of both of these species in phylogenetic analyses was the first time that *S. schenckii* was linked to the ophiostomatoid fungi, providing novel insight into the environmental component of the pathogen’s ecology (Berbee & Taylor 1992).

The three *S. stenoceras* genomes presented here represent the second species from the *S. stenoceras* complex with available genome sequences, alongside *S. protearum* (Du et al. 2022). This increases the number of genomes from environmental *Sporothrix* species to 15. These genomic resources have proven invaluable in the study of other fungal pathogens, with comparisons between environmental and clinical strains providing great insight into the acquisition or loss of pathogenicity, and the evolutionary mechanisms underlying these physiological differences (Pryszcz et al. 2013; Desjardins et al. 2017; Horta et al. 2022)

(*Authors*: Taygen Fuchs*, Deanné du Plessis, Chanel Thomas, Ariska van der Nest, Alishia van Heerden, Brenda D. Wingfield, and Michael J. Wingfield

*Contact: taygen.fuchs@fabi.up.ac.za).

### Supplementary Information

Supplementary Material 1.

Supplementary Material 2.

Supplementary Material 3.

Supplementary Material 4.

Supplementary Material 5.
