# Supplementary material for: IMA Genome – F19: A genome assembly and annotation guide to empower mycologists, including annotated draft genome sequences of Ceratocystis pirilliformis, Diaporthe australafricana, Fusarium ophioides, Paecilomyces lecythidis, and Sporothrix stenoceras
Source: IMA Fungus. 2024 Jun 3;15:12. doi: 10.1186/s43008-024-00142-z (PMC11149380; doi:10.1186/s43008-024-00142-z)
Supplement: Supplementary file 2 — Supplementary Material 2. [file 43008_2024_142_MOESM2_ESM.docx]

**File S1: Step-by-Step Genome Assembly** **and Annotation**

By Janneke Aylward & Andi Wilson

#

# Contents

[Some conventions and suggestions](#_Toc150421317)

[Important disclaimers](#_Toc150421318)

[Programs used in this guide](#_Toc150421319)

[Databases used in this guide](#_Toc150421320)

[Initial assembly](#_Toc150421321)

[Confirm the species identity of your assembly](#_Toc150421322)

[BlobToolKit (BTK) screening](#_Toc150421323)

[Estimate coverage depth and identify mitochondrial contigs](#_Toc150421324)

[Finalise the assembly and submit to GenBank](#_Toc150421325)

[Repeat Masking and gene prediction](#_Toc150421326)

[Functional annotation](#_Toc150421327)

# Some conventions and suggestions

- A greater-than sign (“>”) and Courier New font denote commands. *Do not include the first “>” in your command*
- The names of files or directories specified within a pair of less-than/greater-than signs (i.e. <file>) are files/folders that you can edit/name according to your own needs or naming practices. *Do not include the <> in the name*
- Paths specified within a pair of less-than/greater-than signs (i.e. <path/to/database>) will be specific to your system
- Commands that run over multiple lines are indicated with \
- If using a remote server, we recommend that you run commands in screen (screen -S <name>) and use Ctrl+A+D to exit the screen while leaving the command running in the background. Re-enter the screen using screen -r <name>
- Delete superfluous screens using screen -XS <name> quit
- Check what screens are running with screen -r
- All references to the number of threads/nodes/cores/CPUs to use for a specific command are guides and should be evaluated based on the capacity and availability of the server you are working on
- Take note of the program versions you are using

# Important disclaimers

1. It is beyond the scope of this document to explain basic Linux use and program installation. Linux resources are abundant and the documentation of each program sets out its specific installation process. As such, we assume in this guide that the user has basic Linux knowledge and access to a platform on which these programs are already available.
2. This is an example workflow, and the user should be aware that multiple alternative programs are available for each step. Although we have employed these programs extensively in our own research, we do not claim that they are superior to others or that they are always the most suited to a specific task. This is specifically important to note in the case of contamination screening: while we present the use of BlobToolKit, such in depth screening may be superfluous for many applications. We encourage the user to consider the specific requirements of their project and to choose an appropriate workflow accordingly.

# Programs used in this guide

Listed in order of use:

- FastQC (<https://www.bioinformatics.babraham.ac.uk/projects/fastqc/>)
- Trimmomatic (<http://www.usadellab.org/cms/?page=trimmomatic>)
- SPAdes (<https://github.com/ablab/spades>)
- QUAST (<https://quast.sourceforge.net/>)
- BUSCO (<https://busco.ezlab.org/>)
- SeqKit (<https://bioinf.shenwei.me/seqkit/>)
- Bowtie2 (<https://github.com/BenLangmead/bowtie2>)
- Samtools (<https://github.com/samtools/samtools>)
- BLASTn (<https://ftp.ncbi.nlm.nih.gov/blast/executables/blast+/LATEST/>)
- DIAMOND (<https://github.com/bbuchfink/diamond>)
- BlobToolKit (<https://github.com/blobtoolkit/blobtoolkit>)
- Funannotate (<https://github.com/nextgenusfs/funannotate>)
- RepeatModeler (<https://github.com/Dfam-consortium/RepeatModeler>)
- RepeatMasker (<https://www.repeatmasker.org/>)
- EggNOG-Mapper (<https://github.com/eggnogdb/eggnog-mapper>)
- Interproscan (<https://github.com/ebi-pf-team/interproscan>)
- antiSMASH (<https://github.com/antismash/antismash>)
- Phobius (<https://phobius.sbc.su.se/data.html>)

# Databases used in this guide

Only databases not automatically downloaded during program installation are listed

- BUSCO v4 datasets specific to the study organism, do not necessarily have to be pre-loaded (see step 7); <https://busco.ezlab.org/busco_v4_data.html>
- NCBI “nt” database; <https://ftp.ncbi.nlm.nih.gov/blast/db/>
- NCBI Taxdump; <https://ftp.ncbi.nlm.nih.gov/pub/taxonomy/>
- UniProt database; <http://ftp.ebi.ac.uk/pub/databases/uniprot/current_release/knowledgebase/reference_proteomes/>

# Initial assembly

1. **QC raw reads**
   1. **FastQC**

Assesses the quality of the individual sequence reads and gives you an idea of what kind of filtering/trimming you need to do. The output files will be in .html format and will need to be downloaded and viewed in an internet browser. The FastQC report files include various tables and graphs [**summarising**](https://nf-co.re/eager/2.3.2/docs/output#fastqc) the quality of the sequence reads. Here are examples of [**good**](https://www.bioinformatics.babraham.ac.uk/projects/fastqc/good_sequence_short_fastqc.html#M9) and [**bad**](https://www.bioinformatics.babraham.ac.uk/projects/fastqc/bad_sequence_fastqc.html#M9) quality libraries.

[**FastQC Usage**](https://hbctraining.github.io/Intro-to-rnaseq-hpc-salmon-flipped/lessons/05_qc_running_fastqc_interactively.html#:~:text=FastQC%20provides%20a%20simple%20way,on%20to%20the%20next%20analysis.)

> mkdir <fastqc_out>

> fastqc <file_R1.fastq.gz> <file_R2.fastq.gz> -o <fastqc_out>

1. **Trim raw reads**
   1. **Trimmomatic**

Trims and filters your reads based on information you provide, including quality cutoffs, read lengths and a fasta file with known sequence adapters*.

[**Trimmomatic Usage**](http://www.usadellab.org/cms/uploads/supplementary/Trimmomatic/TrimmomaticManual_V0.32.pdf)

* Make sure that you included the relevant adapter file in the directory in which you are running Trimmomatic so that the adapters are remove. In our case, this was the TruSeq3-PE-2.fa file.

> java -jar $TRIMMOMATIC PE -phred33 -threads 2 \

<file_R1.fastq.gz> <file_R2.fastq.gz> \

<file_R1_trimmed.fastq.gz> <file_R1_singles.fastq.gz \

<file_R2_trimmed.fastq.gz> <file_R2_singles.fastq.gz> \

ILLUMINACLIP:TruSeq3-PE-2.fa:2:30:10 \

LEADING:20 TRAILING:20 SLIDINGWINDOW:4:25 MINLEN:50 \

2>&1 | tee trimmomatic.log

- 1. Combine the two trimmed single files into one file

> cat <R1_singles.fastq.gz> <R2_singles.fast.gz> \

> <Singles.fastq.gz>

1. **Redo FastQC**

Perform this step again on your *trimmed* reads to confirm that Trimmomatic has successfully trimmed poor quality reads and removed the adapters.

1. **Assemble raw reads**
   1. **SPAdes**

Uses the trimmed paired-end and single reads to assemble the genome into scaffolds and/or contigs. Unless you have a sequence library to facilitate additional scaffolding (e.g. a mate-pair library), the two output files (contigs.fasta and scaffolds.fasta) will likely be identical.

[**SPAdes Usage**](https://cab.spbu.ru/files/release3.12.0/manual.html)

> spades.py -t 4 -o <StrainNumber_spades> \

--pe1-1 <R1_trimmed.fastq.gz> \

--pe1-2 <R2_trimmed.fastq.gz> \

--pe1-s <Singles.fastq.gz>

**Output = assembly v1.0**

1. **Filter assembly v1.0 for length (*at this stage use 500 bp*)**
   1. **Seqkit**

A very useful toolkit for fasta and fastq data manipulation. The “seq” flag can be used to transform sequences (eg: filtering, extracting read IDs, removing gaps etc). Filter out any scaffolds less than 500 bp as they are not particularly informative.

[**Seqkit Usage**](https://bioinf.shenwei.me/seqkit/usage/)

> seqkit seq -m 500 scaffolds.fasta > <assembly1.1.fasta>

**Output = assembly v1.1**

1. **Compute statistics of assembly v1.1**
   1. **QUAST**

Calculates basic quality statistics for the assembly, including size, number of scaffolds, GC content, [N50 and L50](https://www.molecularecologist.com/2017/03/29/whats-n50/#:~:text=While%20N50%20corresponds%20to%20the,of%20confusion%20to%20these%20metrics.).

[**QUAST Usage**](https://quast.sourceforge.net/docs/manual.html)

> quast.py -o <Quast> -t 4 <assembly1.1.fasta>

1. **Estimate assembly v1.1 completeness**
   1. **BUSCO**

Assesses genome completeness by comparing the genes present in the genome to evolutionarily informed expectations of gene content from near-universal single-copy orthologs. In other words, it looks for genes known to be universal in a particular group of organisms (*eg: fungi, ascomycetes, bacteria*) and determines how complete the assembly is based on how many of these universal genes (“BUSCOs”) are present.

[**BUSCO Usage**](https://busco.ezlab.org/busco_userguide.html)

We recommend using three lineage datasets: bacterial, fungal and whichever class lineage is applicable to your organism. The bacterial lineage is used to assess contamination in the genome assembly.

Note: The command below is set up to use the --offline option and requires that the specific odb10 dataset is [already downloaded](https://busco.ezlab.org/frames/fungi.htm) and specified with --download_path. If using a server with an internet connection, these two flags can be omitted and the dataset will be downloaded automatically.

> busco --offline -i <assembly1.1.fasta> \

--download_path <path/to/busco_downloads> \

-l fungi_odb10 \

-o <busco_fungi> -m genome

**Other lineage options:**

- bacteria_odb10
- ascomycota_odb10
- eurotiomycetes_odb10
- sordariomycetes_odb10
- [Full list](https://busco.ezlab.org/list_of_lineages.html)

# Confirm the species identity of your assembly

1. **Extract the appropriate barcode region(s)**
   1. The aim of this step is to confirm that the genome you have sequenced represents your target species and thus the sequence(s) necessary for taxonomic verification need to be extracted from the assembly itself. In other words, do not use sequences from a PCR done on the genome isolate prior to sequencing.
   2. The relevant sequences can be identified in the genome assembly with local BLAST searches using [CLC](https://digitalinsights.qiagen.com/products-overview/discovery-insights-portfolio/analysis-and-visualization/qiagen-clc-main-workbench/) or [Geneious](https://www.geneious.com/) or the online [blastn suite](https://blast.ncbi.nlm.nih.gov/Blast.cgi?PROGRAM=blastn&BLAST_SPEC=GeoBlast&PAGE_TYPE=BlastSearch)
2. **Gather the appropriate reference sequences from NCBI**
   1. Determine which strains and species need to be included in the phylogenetic tree (remember to include the appropriate type strain for your species) and obtain the sequence data
3. **Construct the phylogenetic tree**
   1. Use your preferred phylogenetics workflow for this step
   2. A relatively simple option that can be considered is the [NGPhylogeny.fr](https://ngphylogeny.fr/) platform. There are various options ranging from “one click” automatic workflows to an “*a la carte*” option that allows you to create your own custom workflow.

# BlobToolKit (BTK) screening

BlobToolKit is a program that enables “[interactive quality assessment of genome assemblies](https://doi.org/10.1534/g3.119.400908)” by visualising the assembly on a taxon-annotated GC-coverage plot (BlobPlot). This step is not necessarily a requirement for each genome assembly project and the user should evaluate its benefits against the computational resources necessary to generate the BLAST output. In most cases, it should be sufficient to filter for sequence length (as described in step 5) and coverage (see notes under step 17b).

To run BTK on a genome assembly, BTK requires data about the (i) coverage depth and (ii) the taxonomic identity of each contig in the assembly. The first is provided in the form of a BAM file that we can generate with an appropriate mapping algorithm (step 11). For the second, we use the text files output by BLASTn and/or DIAMOND BLASTx (step 12). It is also possible to view a BlobPlot of your assembly without adding BLAST data – see [Issue #181](https://github.com/blobtoolkit/blobtoolkit/issues/181) on the [BTK Github](https://github.com/blobtoolkit/blobtoolkit) page for an explanation.

1. **Map trimmed reads to assembly v1.1**

In this guide, we use [Bowtie2](https://github.com/BenLangmead/bowtie2), a mapper that is efficient at aligning sequences of 50-1000 bp to a reference sequence. There are many mapping algorithms and you may need to choose a different one, depending on the length of your sequence reads.

[**Bowtie2 Usage**](https://bowtie-bio.sourceforge.net/bowtie2/manual.shtml)

- 1. **Bowtie2: indexing**

Before mapping, the mapper needs to build an index of the genome assembly. Like the index of a book, an index of a large DNA sequence enables shorter sequences embedded within it to be found rapidly.

> bowtie2-build <assembly1.1.fasta> <index_prefix>

- 1. **Bowtie2: mapping**

Maps the sequence reads to the index. Use the trimmed reads for best results. See the [manual](https://bowtie-bio.sourceforge.net/bowtie2/manual.shtml) for a description of the input options.

> bowtie2 --threads 4 -x <index_prefix> \

-1 <file_R1_trimmed.fastq.gz> -2 <file_R2_trimmed.fastq.gz> \

-U <Singles.fastq.gz> -S <out.sam>

- 1. **Samtools: Convert to bam, sort and index**

Mapping data is typically generated as [Sequence Alignment Map (SAM)](https://en.wikipedia.org/wiki/SAM_(file_format)) formatted files. Depending on the amount of data they hold, these text files can be several GB large and should be compressed. We use samtools to convert SAM files to [Binary Alignment Map (BAM)](https://en.wikipedia.org/wiki/Binary_Alignment_Map) format and then sort and index the BAM file to enable other programs to rapidly retrieve alignment data.

Indexes can have different formats. In our case, BTK requires a .csi index file, therefore, we specify the “-c” flag during indexing.

[**Samtools Usage**](http://www.htslib.org/doc/samtools.html)

> samtools view <out.sam> -b -o <out.bam>

> samtools sort <out.bam> -o <sorted.bam>

> samtools index -c <sorted.bam>

- 1. **Delete redundant files**

After generating the sorted BAM file, the SAM (out.sam) and unsorted BAM (out.bam) files are no longer needed. It is important to delete them because these intermediate files can be VERY large. Be careful that you do not delete your final sorted bam file.

> rm <out.sam> <out.bam>

1. **Assign taxonomy information to each contig in assembly v1.1**
   1. [Command-line BLAST](https://www.ncbi.nlm.nih.gov/books/NBK279690/) is highly versatile and useful for running large batch jobs. In this case, we use it to assign a putative taxonomic origin to each contig in the assembly. [Many different output formats](https://www.ncbi.nlm.nih.gov/books/NBK279684/table/appendices.T.options_common_to_all_blast/) are possible and the tabular outputs are customizable. BTK requires [specific BLAST output formats and parameters](https://blobtoolkit.genomehubs.org/blobtools2/blobtools2-tutorials/getting-started-with-blobtools2/#add_blast) and the commands below are, therefore, based on these requirements.
   2. **BLASTn**

Compares each sequence against the NCBI “nt” database. This database has to be downloaded to perform a local BLAST search because the NCBI server does not allow such large jobs to run remotely (i.e. the -remote option will not work).

> blastn \

-db <path/to/your/local/download/of/the/NCBI/nt/database> \

-query <assembly1.1.fasta> \

-outfmt "6 qseqid staxids bitscore std" \

-max_target_seqs 10 -max_hsps 1 -evalue 1e-25 \

-out <blast.out>

- 1. **DIAMOND BLASTx**

Translates each sequence into all six reading frames and compares it against the UniProt Knowledgebase ([UniProtKB](https://www.uniprot.org/help/uniprotkb); extensively curated protein information). [Diamond](https://github.com/bbuchfink/diamond) is sequence aligner built for large datasets and is “100x-10,000x” faster than BLAST.

> diamond blastx \

--db <path/to/uniprot/reference_proteomes.dmnd> \

--outfmt 6 qseqid staxids bitscore qseqid sseqid pident length mismatch gapopen qstart qend sstart send evalue bitscore \

--query <assembly1.1.fasta> \

--sensitive --max-target-seqs 1 --evalue 1e-25 \

--threads 4 > <diamond.blastx.out>

1. **Create and populate the BTK directory (“BlobDir”)**

Once the necessary files have been prepared, all the data pertaining to the genome assembly is gathered into a single directory called the BlobDir. In the first step (a), this directory is created and the taxonomic identity of the organism in question is optionally provided. The second step (b), populates this directory with the available data.

- 1. **Create the BlobDir**

The --taxid and --taxdump fields are optional (but if --taxid is used, --taxdump is required). Find the Taxonomy ID of your species at <https://www.ncbi.nlm.nih.gov/taxonomy>.

[**BlobTools create Usage**](https://blobtoolkit.genomehubs.org/blobtools2/blobtools2-tutorials/getting-started-with-blobtools2/#create_blobdir)

> blobtools create --fasta <assembly1.1.fasta> \

--taxid <NCBI_taxid_number_of_your_species> \

--taxdump <path/to/downloaded/NCBI/taxdump/> \

<BlobDir_name>

- 1. **Populate the BlobDir**

In addition to the coverage and taxonomic data, BUSCO data (generated in step 7) can also be added to the BlobDir, although this is optional. The results of multiple BUSCO lineages can be included by including a --busco line for each lineage.

Data can also be added to the BlobDir in multiple cycles, e.g. you can run blobtools add with only the --cov option and add BLAST and BUSCO data in the next cycle. Add the --replace flag to overwrite one dataset version (of e.g. BUSCO) with another.

Remember that the taxdump database must be specified whenever you add taxonomy information.

[**Blobtools add Usage**](https://blobtoolkit.genomehubs.org/blobtools2/blobtools2-tutorials/adding-data-to-a-dataset/)

> blobtools add \

--cov <sorted.bam> \

--busco <busco_fungi>/run*/full_table.tsv \

–-busco <busco_bacteria>/run*/full_table.tsv \

--hits <blast.out> --hits <diamond.blastx.out> \

--taxdump <path/to/downloaded/NCBI/taxdump/> \

<BlobDir_name>

1. **View the BlobPlot**
   1. **Host the parent directory of the BlobDir**

To view the BlobPlot, you need to host the directory within which the BlobDir is situated (e.g. if the path to your BlobDir is /users/janedoe/btk/blobdir, you need to host the btk directory). Do this by running blobtools host from the directory one level up from the parent directory (in this example janedoe) or supplying the full path to the parent directory (/users/janedoe/btk).

It is also important that the parent directory contains ***only*** one or more BlobDirs and no other files or folders. If there are other things, blobtools host often only shows a white screen.

The blobtools host command below specifies the default API port and HTTP port numbers. You will need to change these if multiple users are on the system at the same time. You will know this is the case if you get an error from blobtools host stating that those ports are in use.

If you are running BTK on your local machine, you do not need to specify port numbers or a hostname.

[**Blobtools host Usage**](https://blobtoolkit.genomehubs.org/blobtools2/blobtools2-tutorials/opening-a-dataset-in-the-viewer/)

> blobtools host \

--api-port 8000 --port 8080 \

--hostname <remote.server.address> \

<directory>

- 1. **Open the BlobPlot in a web browser**

While blobtools host is running, go to a web browser and enter this address: http://<remote.server.address>:<port_number>/view/all

[e.g.: <http://imagenaryserver.up.ac.za:8080/view/all>]

1. **Filter the assembly on the BTK Viewer**

[**BTK Viewer guide**](https://blobtoolkit.genomehubs.org/btk-viewer/viewer-tutorials/filtering-assemblies/) [For an in depth explanation of the BTK Viewer, consider [this online course](https://www.futurelearn.com/courses/eukaryotic-genome-assembly-how-to-use-blobtoolkit-for-quality-assessment/)]

- 1. **Contig length**

Based on experience and the typical length of coding sequences, we recommend a minimum contig length of 1000 bp.

- 1. **Coverage depth**

We recommend discarding contigs with a coverage depth that is much lower than the average coverage. The cut-off will be arbitrary and based on your judgement, but it is often intuitive to recognize a cluster (or “blob”) of contigs with a coverage much lower than the main portion of the genome. Additionally, these are often small contigs and/or contigs that could not be assigned a taxonomic identity, implying that they may be low complexity sequences that could not be assembled properly. In other cases, they may be contaminants (see next point). As an example, if your primary “blob” shows an average coverage of around 100x, it would be worth filtering out contigs with less than 20x coverage.

- 1. **Taxonomy**

BTK is based on the premise that sequences from different taxa have inherently different GC contents and that non-target taxa are likely to have a different level of abundance in the starting sample compared to the target organisms (measured in the form of sequence coverage). Non-target taxa will, therefore, likely form their own blobs, separate from the main blob of the target genome. Be careful about filtering contigs that lie within the main blob based only on taxonomic identity because it is possible that the origin of a contig could have been assigned incorrectly (remember that BLAST results depend on what is available in the NCBI databases).

Taxonomic origin becomes less sensitive at lower taxonomic levels so do not filter strictly based on Class or Order level unless you have good reason to do so. For a fungal genome project, we recommend removing contigs with a putative non-fungal origin. Keep contigs labelled “no-hits” - these could not be assigned a taxonomic origin and (once length and coverage filtering has been done) these likely form the non-coding/repetitive portions of the genome that could not be fully assembled.

NB: Copy and save the URL of the final filtered plot because [it contains the information about how the assembly was filtered](https://blobtoolkit.genomehubs.org/btk-viewer/viewer-tutorials/reproducing-interactive-sessions/). It will be used in the next step.

1. **Extract the BTK-filtered assembly from assembly v1.1**
   1. **blobtools filter**

There are multiple ways to filter with this command. Here, we only show how to apply the changes made on the BTK viewer using the URL generated during the previous step. The filtered assembly fasta will be saved in the same directory as the unfiltered assembly from which it is extracted (in this case assembly v1.1) and will have .filtered included in its name.

[**Blobtools filter Usage**](https://blobtoolkit.genomehubs.org/blobtools2/blobtools2-tutorials/filtering-a-dataset/)

> blobtools filter \

--query-string <“URL”> \

--fasta <assembly1.1.fasta> \

<BlobDir_name>

**Output = assembly v2.0**

# Estimate coverage depth and identify mitochondrial contigs

1. **Estimate coverage depth**

[Theoretically](https://doi.org/10.1038/nrg3642), the average coverage depth of a genome assembly is the number of sequenced bases divided by the genome size. Actual read coverage will, however, differ from the theoretical coverage since some sequence data was trimmed during the QC process and not all bases have been sequenced at an equal rate. For any given nucleotide, read coverage or sequencing depth is the number of times that specific nucleotide has been read in the sequencing experiment. This information is found in the SAM/BAM/CRAM mapping file.

- 1. **Using BTK**

After filtering, navigate to the [Table view](https://blobtoolkit.genomehubs.org/btk-viewer/viewer-tutorials/exploring-views/) in the BTK viewer. Export the table as a .csv file (make sure that this is the same version as the filtered assembly v.2.0 that you have just created) and open the table in a spreadsheet program.

Sort this table by coverage and remove outliers. We have already removed low-coverage contigs from the assembly, but high-coverage outliers (such putative mitochondrial sequences) may be present.

Calculate the average and standard deviation of coverage. This can be done for all contigs (excluding outliers) or for a subset of contigs. A good rule of thumb is to calculate coverage based on the largest n=L50 contigs in the assembly, as these have been assembled best and should provide a good estimate of coverage depth. The standard deviation of coverage is not often reported, but it is important to consider. Standard deviation should be low so that one can be confident that the average coverage depth is an accurate reflection of the coverage of the whole assembly.

We recommend reporting coverage depth as a whole number rounded down to the nearest 5 - e.g. 95X, 100X, 105X.

- 1. **Alternative programs to estimate coverage**

Many other programs can also estimate coverage depth from a sorted BAM file, this is by no means an exhaustive list.

- - 1. The [BBMap package](https://jgi.doe.gov/data-and-tools/software-tools/bbtools/bb-tools-user-guide/bbmap-guide/):

> pileup.sh in=<sorted.bam>

- - 1. [Samtools](http://www.htslib.org/doc/samtools-depth.html):

> samtools depth -a <sorted.bam> > <assembly1.1.depth>

The output of samtools depth can be imported into a spreadsheet and can also facilitate filtering based on coverage depth (i.e. if not using BTK). In a text file, make a list of contigs names to keep and then use an appropriate tool (like [bioawk](https://bioinformaticsworkbook.org/Appendix/Unix/bioawk-basics.html#gsc.tab=0) or [faSomeRecords](file:///Users/janneke/Downloads/gaw_20231129/faSomeRecords) to extract those specific sequences).

Take note that, if you are following this workflow, these two (and any other) alternative methods will calculate the depth of all the contigs in assembly v1.1 (not the filtered assembly v2.0 output of BTK). To estimate the coverage of assembly v2.0, you will need to filter the output of these alternative methods and calculate the coverage of a subset of contigs.

1. **Identify putative mitochondrial contigs**

Identifying all mitochondrial contigs and assembling a complete “mitogenome” is beyond the scope of this guide. The aim in this step is to identify any contigs with an obvious mitochondrial origin, using two lines of evidence:

- 1. **Excessive coverage depth**

Eukaryotic cells have [multiple mitochondria per cell](https://doi.org/10.3389%2Ffcell.2016.00085), each containing at least one copy of their own (mito)genome. These sequences, therefore, become overrepresented in the final dataset and have a read coverage that is many orders of magnitude above that of the nuclear genome. Consult your table (from step 17) to identify these sequences.

- 1. **Similarity to other mitochondrial sequences**

If available, download the mitochondrial genomes of three or four species that are closely related to your study organism from GenBank. Perform local BLAST searches against assembly v2.0 to identify putative mitochondrial sequences.

# Finalise the assembly and submit to GenBank

1. **Clean, sort and rename the contigs in assembly v2.0**

Create the final version of the assembly.

[**Funannotate clean and sort Usage**](https://funannotate.readthedocs.io/en/latest/prepare.html)

- 1. **Identify and “clean” up duplicate contigs**

Duplicate contigs are not expected to be present, especially not in haploid genomes. Therefore, this step is not strictly necessary.

> funannotate clean --input <assembly2.0.fasta> \

--out <assembly2.1.fasta>

- 1. **Sort and/or rename FASTA headers**

> funannotate sort --input <assembly2.1.fasta> \

--out <assembly2.1_renamed.fasta> --base Scaffold

**Output = assembly v2.1**

1. **Compute statistics of assembly v2.1**
   1. **Calculate the statistics of the final assembly**
      1. QUAST (see step 6)
      2. BUSCO (see step 7)

If you can see via the BTK Viewer that the BUSCO values are unchanged or that you have eliminated bacterial contamination (if applicable), it is not necessary to rerun BUSCO.

1. **Start a WGS submission**

To prevent the need to redo analyses, we want to confirm that the final version of our assembly is acceptable to GenBank before continuing with gene prediction.

- 1. **Register a BioProject and BioSample**

This can be done during the WGS submission workflow or you can specify previously registered projects, if applicable. For these, as well as the WGS submission itself, we recommend selecting the maximum embargo period.

- 1. **Choose an informative locus_tag**

This is chosen during the BioProject submission. If you do not choose one, NCBI will automatically assign an uninformative code as your locus_tag (this becomes the prefix to all gene predictions). As a personal preference, for *Species hypotheticus* XX63, we would recommend Shyp63.

- 1. **Do not select “automatic finish”**

At this stage, we are confirming that GenBank is happy with your genome assembly, so do not enable this option. After you submit, NCBI will run their [Foreign Contamination Screen (FCS) tool](https://github.com/ncbi/fcs). If you have performed screening with BTK, it is unlikely to detect additional contamination. If it does, filter your assembly according to the instructions. Do not finalise the submission yet.

- 1. **Create an .sbt file**

As a final administrative step, create this file using the template provided at <https://submit.ncbi.nlm.nih.gov/genbank/template/submission/>. It will be used during the final functional annotation step.

1. If GenBank asked for additional filtering of assembly v2.1:
   1. redo funannotate sort (step 19b) to rename contigs in sequential order
   2. if not, continue using assembly v2.1

**Output = assembly v2.2**

# Repeat Masking and gene prediction

1. **Identify repeats in assembly v2.1 (or v2.2, if applicable)**

Repeat identification is the first form of genome annotation that we perform in this guide. It is important to do this before gene prediction because some repeats can interfere with gene prediction algorithms. This is because some interspersed repeats encode their own genes and some algorithms may struggle to predict genes in repeat rich regions. Repeats also complicate BLAST searches and may cause spurious hits.

There is no “best” way to detect repeats: “[A single tool cannot detect all different types of repeats in diverse species](https://doi.org/10.1101/2021.09.10.459798)…” There are a myriad of tools for repeat identification and classification and most modern algorithms are pipelines that use a variety of different tools. Keep in mind that at this stage our goal is not to study genome-wide repeat content, but to identify and mask (hide) repeats that may interfere with gene prediction.

- 1. **RepeatModeler**

RepeatModeler creates a comprehensive custom library of repeat sequences in the genome assembly and also classifies the repeats in the library (the classification step is not necessary for gene prediction, but is a bonus for those interested).

[**RepeatModeler Usage**](https://github.com/Dfam-consortium/RepeatModeler#example-run)

This program does not produce informative log files automatically, so log the screen output with nohup

> BuildDatabase -name <database_prefix> \

<assembly2.1_renamed.fasta>

> nohup RepeatModeler -LTRStruct -pa 5 \

-database <database_prefix> > repmod.log

- 1. **Count the number of repeat families** (for interest’s sake)

The custom repeat library (*families.fa) contains the consensus sequence of each repeat family detected in the assembly. Therefore, the number of fasta entries in this file is the number of repeat families detected in the genome.

> grep -c “>” *families.fa

1. **Mask repeats in assembly v2.1 (or v2.2, if applicable)**
   1. **RepeatMasker**

The repeats identified by RepeatModeler still need to be hidden from the gene prediction algorithms. Hard masking converts all repetitive bases to “N’s” and is, therefore, irreversible. It is better to apply soft-masking (-xsmall option), in which repetitive bases are converted from capital to lowercase letters.

[**RepeatMasker Usage**](https://www.repeatmasker.org/webrepeatmaskerhelp.html)

> RepeatMasker <assembly2.1_renamed.fasta> \

-lib *families.fa -pa 5 -xsmall -gff

- 1. **RepeatMasker** [**output files**](https://www.repeatmasker.org/webrepeatmaskerhelp.html#reading)

Three output files are directly relevant:

- - 1. genome.fasta.masked: the soft-masked assembly to use downstream
    2. genome.fasta.tbl file: the repeat statistics
    3. genome.fasta.gff: can be used to view the position of repeats on the assembly in a genome browser.

**Output = a masked assembly: assembly v3.0**

1. **Predict genes in assembly v3.0**
   1. **Funannotate**

[This program](https://github.com/nextgenusfs/funannotate) was built with the mission to simplify gene annotation and ultimate GenBank submission. It considers both *ab initio* gene predictions as well as BLAST alignments of experimentally verified protein sequences from the UniProtKb/SwissProt curated database. Additionally, user-specified protein or transcript evidence can be supplied. It also uses [tRNAscan-SE](https://doi.org/10.1093/nar/gkab688) to predict tRNA genes.

[**Funannotate predict Usage**](https://funannotate.readthedocs.io/en/latest/predict.html)

Remember to use the locus_tag generated during WGS submission. If you have RNASeq data for your species and/or ESTs and/or curated protein models from closely related species, consult the [funannotate documentation](https://funannotate.readthedocs.io/en/latest/evidence.html) about how to add these data. Do not use uncurated protein evidence.

> funannotate predict -i <assembly3.0.fasta> \

--out <funannotate_dir> \

--species <“Genus epithet”> \

--strain <CMW12345> \

--name <“locus_tag”> --cpus 5

- 1. **View results and count gene models**

The results of this prediction will be in the predict_results directory. Predictions can be viewed in a genome browser using the .gbk output file or a combination of the .gff3 and genome.fasta files.

The funannotate-predict.log file with the logfiles directory will contain the information about how many genes were predicted. You can also directly count the number of entries in the fasta files within predict_results. Remember that the number of entries in *cds-transcipts.fa and *mrna-transcipts.fa will differ because the predicted tRNA genes are included only in *mrna-transcripts.fa.

> grep -c “>” *fa

#

# Functional annotation

The aim of functional annotation is to associate biological information with structural annotations. Functional annotation is inferred from similar sequences with known functions and relies on sequence homology. The quality of the annotation will, therefore, depend on the quality of information in the databases and curated databases are primarily used to assign functional annotations.

We must also consult multiple databases, since databases typically specialise in different functional groups. The list of programs given here is based on what is integrated into the Funannotate functional annotation pipeline and is not exhaustive. Instead of one output for each database, Funannotate collates the results and returns them in a single file. Your specific research goal(s) may lead you to use only some of these databases or include others not mentioned here.

**Funannotate integrates:**

- *BUSCO groups
- *CAZYmes (dbCAN)
- Eggnog annotations and COGs
- InterProScan5 (InterPro terms, GO ontology, and fungal transcription factors)
- *Pfam domains
- *Proteases (MEROPS)
- *Secreted proteins (SignalP)
- Secondary metabolite biosynthesis clusters (antismash)
- Transmembrane proteins (Phobius)

Some of these programs (marked with *) can be run automatically within the funannotate annotate pipeline, whereas the rest need to be run separately and the output files provided to funannotate. Wrapper scripts for Interpro, Antismash and Phobius exist within Funannotate, but these can be slow and often fail when something is changed/updated on the third-party’s side.

Of the stand-alone programs, funannotate annotate only insists on Eggnog-Mapper and will skip the others if their outputs are not provided.

1. **EggNOG-Mapper**

Uses precomputed orthologous groups and phylogenies from the [eggNOGdatabase](http://eggnog5.embl.de/) to transfer functional information. Because it is based on similarity to an orthologous group and not individual sequences, it has [higher precision](http://eggnog5.embl.de/#/app/methods) than traditional homology searches (i.e. BLAST).

[**Emapper Usage**](https://github.com/eggnogdb/eggnog-mapper/wiki/eggNOG-mapper-v2.1.5-to-v2.1.11%20-%20basic-usage)

If running eggnog from within annotate_misc, do not use “eggnog” as the <outfile_prefix> because funannotate will overwrite this file. --cpu 0 will use all available cpus: use with caution or change to an appropriate value.

> emapper.py \

-i ../predict_results/*proteins.fa \

--data_dir <path/to/eggnogmapper/data/> \

-o <outfile_prefix> \

--cpu 0

1. **Interproscan**

Classifies proteins into families and predicts domains and important sites, and integrates signatures from [13 member databases](https://www.ebi.ac.uk/interpro/about/consortium/).

[**Interproscan Usage**](https://interproscan-docs.readthedocs.io/en/latest/HowToRun.html)

You can run Interproscan from within the annotate_misc directory within <funannotate_dir>

> interproscan.sh \

-i ../predict_results/*proteins.fa \

--outfile <iprscan.xml> \

--formats xml --cpu 5

1. **antiSMASH**

Genome-wide identification, annotation and analysis of secondary metabolite biosynthesis gene clusters.

- 1. Run the stand-alone version from within <funannotate_dir>

[**antiSMASH Usage**](https://docs.antismash.secondarymetabolites.org/command_line/)

> antismash --taxon fungi --output-dir <antismash> \

--genefinding-tool none --cpus 5 \

predict_results/*.gbk

- 1. Antismash can also be run on the [web server](https://fungismash.secondarymetabolites.org/)

In this case, download the output, unzip and place it in <funannotate_dir> (not in annotate_misc)

1. **Phobius**

“[A combined transmembrane topology and signal peptide predictor](https://phobius.sbc.su.se/instructions.html)”.

[Download](https://phobius.sbc.su.se/data.html) Phobius and unpack the archive, then run Phobius from the annotate_misc directory.

> tar -zxvf phobius101_linux.tgz

> phobius.pl -short \

../predict_results/*proteins.fa \

> phobius.results.txt

The output of Phobius has to be placed in annotate_misc and be named “phobius.results.txt” for funannotate annotate to recognize it

1. **Combine all functional annotations**

Funannotate will parse the results of your input files and run the additional functional annotation steps (indicated with asterisks above). Phobius results will be detected automatically, if named correctly. The annotate script can be run more than once (e.g. to add data). It saves the analyses each time and picks up where it left off.

[**Funannotate annotate Usage**](https://funannotate.readthedocs.io/en/latest/annotate.html)

> funannotate annotate -i <funannotate_dir> \

--iprscan <funannotate_dir/annotate_misc/iprscan.xml> \

--antismash <funannotate_dir/antismash/antismash.gbk> \

--eggnog <funannotate_dir/eggnog.emapper.annotations> \

--cpus 5 --sbt yourspecies.sbt

The output in annotate_results will contain the same files found in predict_results, but these will also have functional information associated with each gene. Files necessary for NCBI submission (.agp; .tbl; .sqn) are also provided, as well as a text file containing the functional information about each predicted gene.

1. **Genome comparisons**

If you are working with more than one genome assembly, you may want to try funannotate compare. Example outputs of this script is provided in File S2.

[**Funannotate compare Usage**](https://funannotate.readthedocs.io/en/latest/compare.html)

> funannotate compare \

-i <funannotate_dir1> <funannotate_dir2> <funannotate_dir3>

1. **Submit annotations to NCBI**

Finally, you can upload your annotations to NCBI by replacing the fasta file in your WGS submission with the .sqn output file* in the annotate_results directory. You might need to contact [genomes@ncbi.nlm.nih.gov](mailto:genomes@ncbi.nlm.nih.gov) and ask them to enable the “Fix” button in order to do this.

*The .sqn file is generated by NCBI’s [tbl2asn](https://www.ncbi.nlm.nih.gov/genbank/tbl2asn2/) script that has been incorporated into Funannotate’s predict and annotate pipelines. NCBI recently replaced this script with [table2asn](https://www.ncbi.nlm.nih.gov/genbank/table2asn/), but is (for the moment) still accepting the tbl2asn output produced by Funannotate. [A future release of Funannotate will incorporate the update](https://github.com/nextgenusfs/funannotate/issues/912).
