# Supplementary material for: IMA Genome – F19: A genome assembly and annotation guide to empower mycologists, including annotated draft genome sequences of Ceratocystis pirilliformis, Diaporthe australafricana, Fusarium ophioides, Paecilomyces lecythidis, and Sporothrix stenoceras
Source: IMA Fungus. 2024 Jun 3;15:12. doi: 10.1186/s43008-024-00142-z (PMC11149380; doi:10.1186/s43008-024-00142-z)
Supplement: Supplementary file 3 — Supplementary Material 3. [file 43008_2024_142_MOESM3_ESM.docx]

File S2: Comparisons of genomes published in IMA-Genome F18

# Summary

Twelve strains belonging to five different fungal species are presented in this issue of IMA-Genome. This is the first IMA-Genome issue in which comparisons are made among the genomes of different species presented in each subsection and is not intended to represent a new standard format. The fact that all genome assemblies in this issue have been assembled and annotated in the same way facilitated comparison among them and use of the Funannotate pipeline further eased this task. The output of the Funannotate compare script is provided here as a summary of the genome assemblies presented in this issue as well as an illustration of the value that gene prediction and functional annotation adds to genome assemblies.

**Table S2-1** shows the assembly and gene prediction statistics for each strain, as well as the results of an orthology analysis among all strains. Genome sizes ranged from 26 Mb in *Ceratocystis pirilliformis* to more than 50 Mb in *Diaporthe australafricana*. The small genome of *C. piriliformis* was also reflected in its low number of predicted genes compared to the other four species. The two species with the largest genomes, *D. australafricana* and *Fusarium ophioides*, had more than twice the number of predicted genes compared to *C. pirilliformis.* More than 95% of the predicted proteins in each strain had at least one ortholog in another strain across all 12 species, but due to the large variation in gene content among the species, only 25-60% of single-copy orthologs were shared among all 12 strains.

The trend seen in the genome size and number of predicted proteins was mirrored in the number of Carbohydrate Active Enzyme (CAZyme; **Figure S2-1 and S2-2)** and peptidase (**Figure S2-3 and S2-4)** protein families that were identified in each genome. In both cases *D. australafricana* had the highest, *F. ophioides* the second highest and *C. piriliformis* the lowest number of predicted genes. The InterProScan domains (**Figure S2-5a**) also clearly separated *C. piriliformis* from the other strains, while analysis of the PFAM domains (**Figure S2-5b**) additionally showed separation of *D. australafricana* and *F. ophioides*.

The final comparison presented here is the number of Transcription Factors identified in each genome (**Figure S2-6**). Of the transcription factor domains identified, two fungal-specific transcription factors, IPR001138 and IPR007219, were by far the most abundant in all 12 genomes. Despite *D. australafricana* having the largest genome assembly and highest number of predicted genes, *F. ophioides* and *Sporothrix stenoceras* had the highest number of transcription factor domains, specifically with regards to the two fungal-specific domains.

## Genome assembly statistics

# **Table S2-1.** Assembly, prediction and orthology statistics of the 12 genomes presented in this issue.

|  | **Assembly** | | | | |  | **Gene-prediction** | | |  | **Orthologous proteins** | | |
| --- | --- | --- | --- | --- | --- | --- | --- | --- | --- | --- | --- | --- | --- |
| **Species and strain** | **Size (bp)** | **Scaffolds** | **Largest Scaffold (bp)** | **N50 (bp)** | **GC%** |  | **Genes** | **Proteins** | **tRNA** |  | **Unique** | **At least 1 ortholog** | **Single-copy orthologs** |
| ***Diaporthe australafricana*** |  |  |  |  |  |  |  |  |  |  |  |  |  |
| CMW-IA:616 | 50,801,140 | 505 | 691,901 | 251,366 | 53.37 |  | 14,572 | 14,404 | 168 |  | 301 | 14,098 | 3,901 |
| CMW-IA:644 | 50,851,703 | 437 | 915,897 | 268,244 | 53.28 |  | 14,527 | 14,374 | 153 |  | 319 | 14,047 | 3,901 |
| ***Fusarium ophioides*** |  |  |  |  |  |  |  |  |  |  |  |  |  |
| CMW-IA:5007 | 44,515,435 | 323 | 1,331,646 | 417,843 | 48.66 |  | 15,047 | 14,742 | 305 |  | 133 | 14,607 | 3,901 |
| CMW-IA:5006 | 44,261,214 | 259 | 1,262,000 | 379,241 | 48.73 |  | 15,044 | 14,740 | 304 |  | 141 | 14,599 | 3,901 |
| CMW-IA:4746 | 43,506,516 | 185 | 1,831,960 | 432,242 | 48.75 |  | 14,361 | 14,059 | 302 |  | 225 | 13,829 | 3,901 |
| ***Sporothrix stenoceras*** |  |  |  |  |  |  |  |  |  |  |  |  |  |
| CMW-IA:5364 | 39,563,291 | 139 | 1,883,983 | 563,416 | 52.79 |  | 10,612 | 10,397 | 215 |  | 76 | 10,320 | 3,901 |
| CMW-IA:5313 | 39,575,170 | 155 | 1,376,613 | 447,009 | 52.79 |  | 10,614 | 10,400 | 214 |  | 96 | 10,302 | 3,901 |
| CMW-IA:5347 | 39,281,339 | 165 | 1,345,308 | 451,359 | 52.90 |  | 10,473 | 10,258 | 215 |  | 98 | 10,158 | 3,901 |
| ***Paecilomyces lecythidis*** |  |  |  |  |  |  |  |  |  |  |  |  |  |
| CMW-IA:5739 | 31,272,536 | 88 | 2,140,311 | 719,368 | 48.55 |  | 9,811 | 9,646 | 165 |  | 415 | 9,215 | 3,901 |
| CMW-IA:550 | 30,958,428 | 98 | 2,528,076 | 674,067 | 48.74 |  | 9,735 | 9,578 | 157 |  | 363 | 9,197 | 3,901 |
| ***Ceratocystis piriliformis*** |  |  |  |  |  |  |  |  |  |  |  |  |  |
| CMW-IA:4944 | 26,159,417 | 637 | 353,044 | 78,262 | 48.10 |  | 7,003 | 6,654 | 349 |  | 98 | 6,553 | 3,901 |
| CMW-IA:5519 | 26,202,346 | 567 | 404,750 | 90,938 | 48.09 |  | 7,012 | 6,657 | 355 |  | 94 | 6,558 | 3,901 |

#

# CAZymes


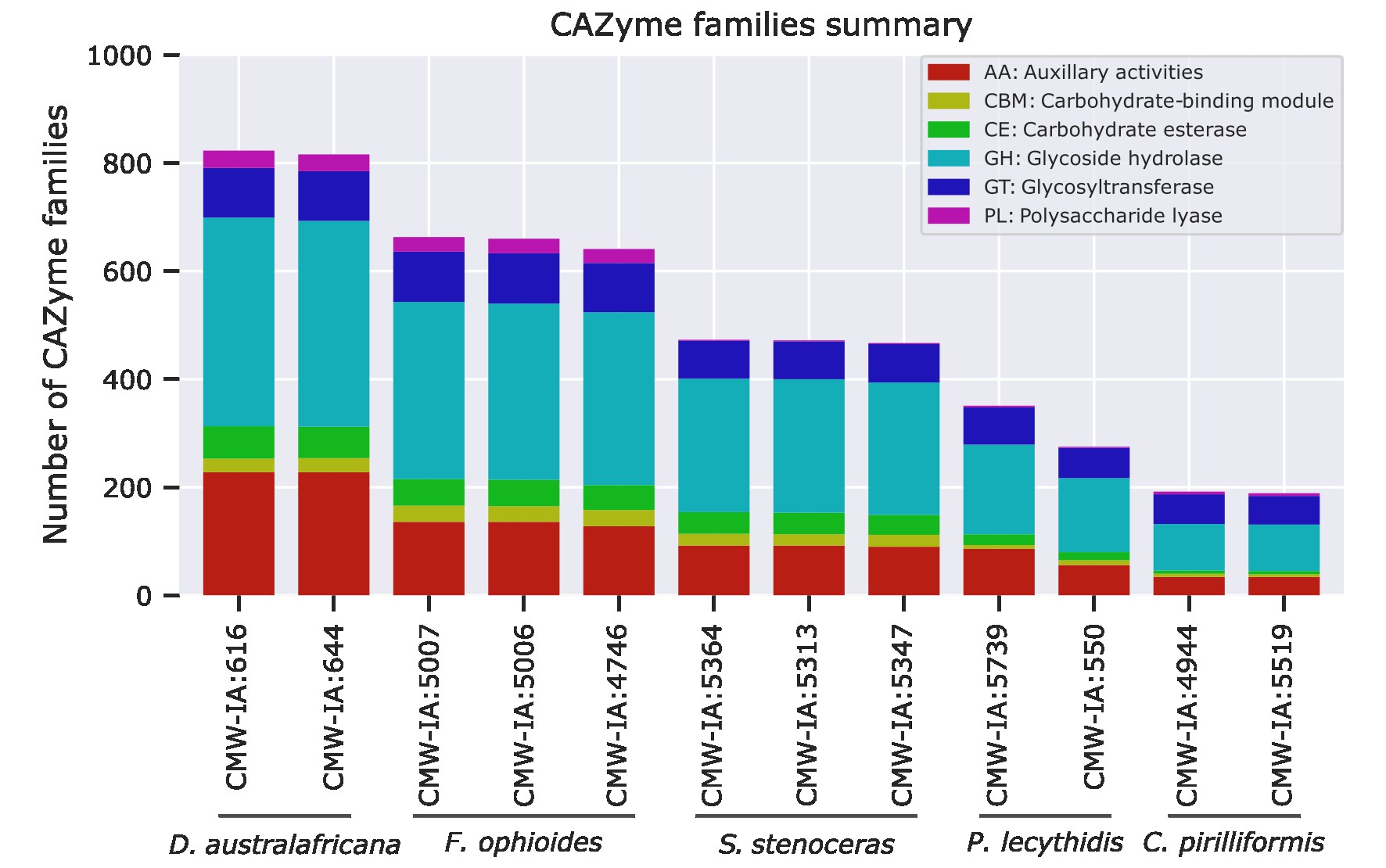


**Figure S2-1.** The number and types of CAZyme (Carbohydrate Active Enzyme) families identified in the predicted proteomes of each strain.


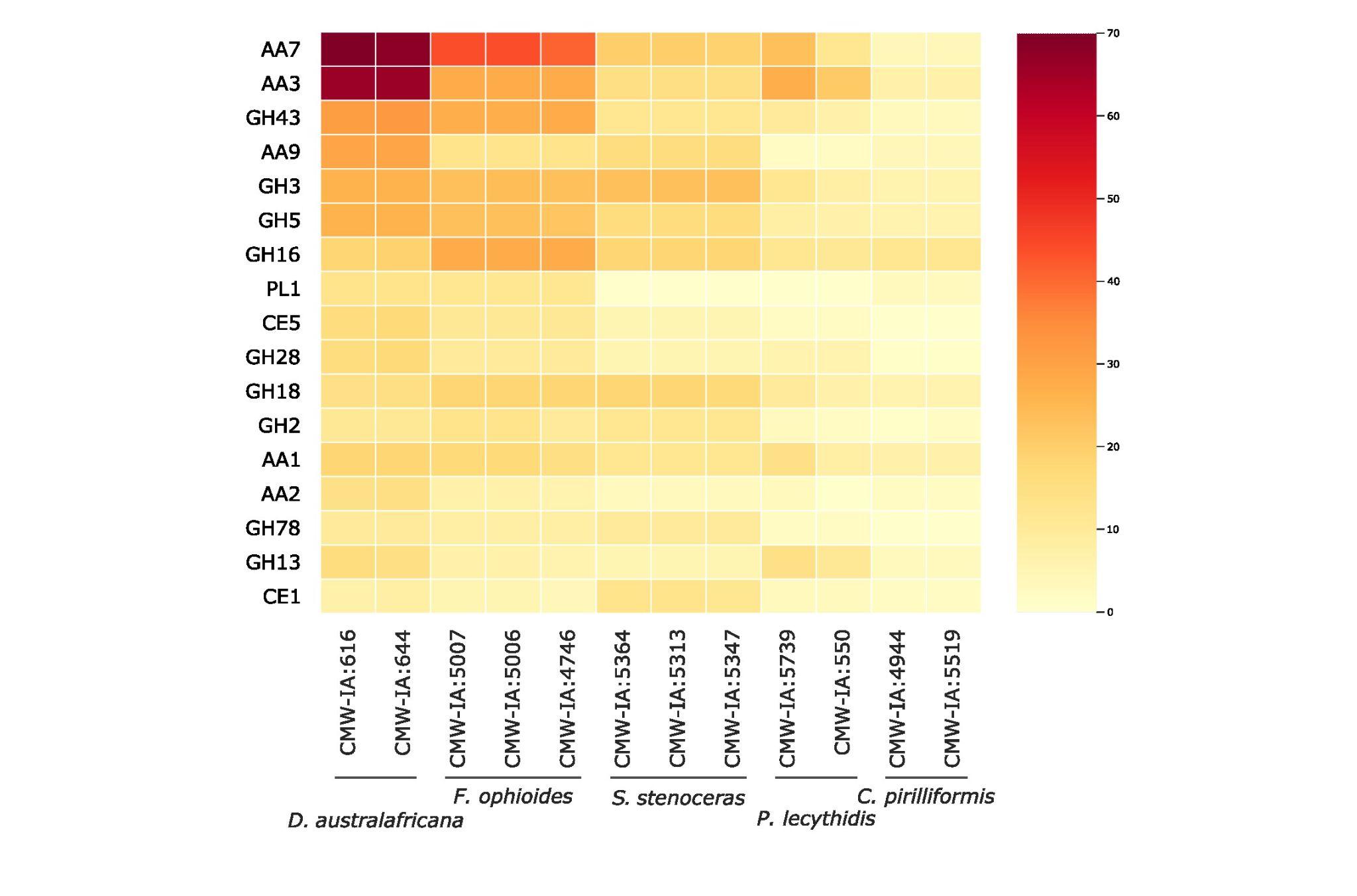


**Figure S2-2.** Heatmap illustrating the differences in the number of CAZyme (Carbohydrate Active Enzyme) families identified in the predicted proteomes of each strain. Only CAZyme families with copy number variation exceeding a standard deviation of four are shown (in descending order).

# Peptidases


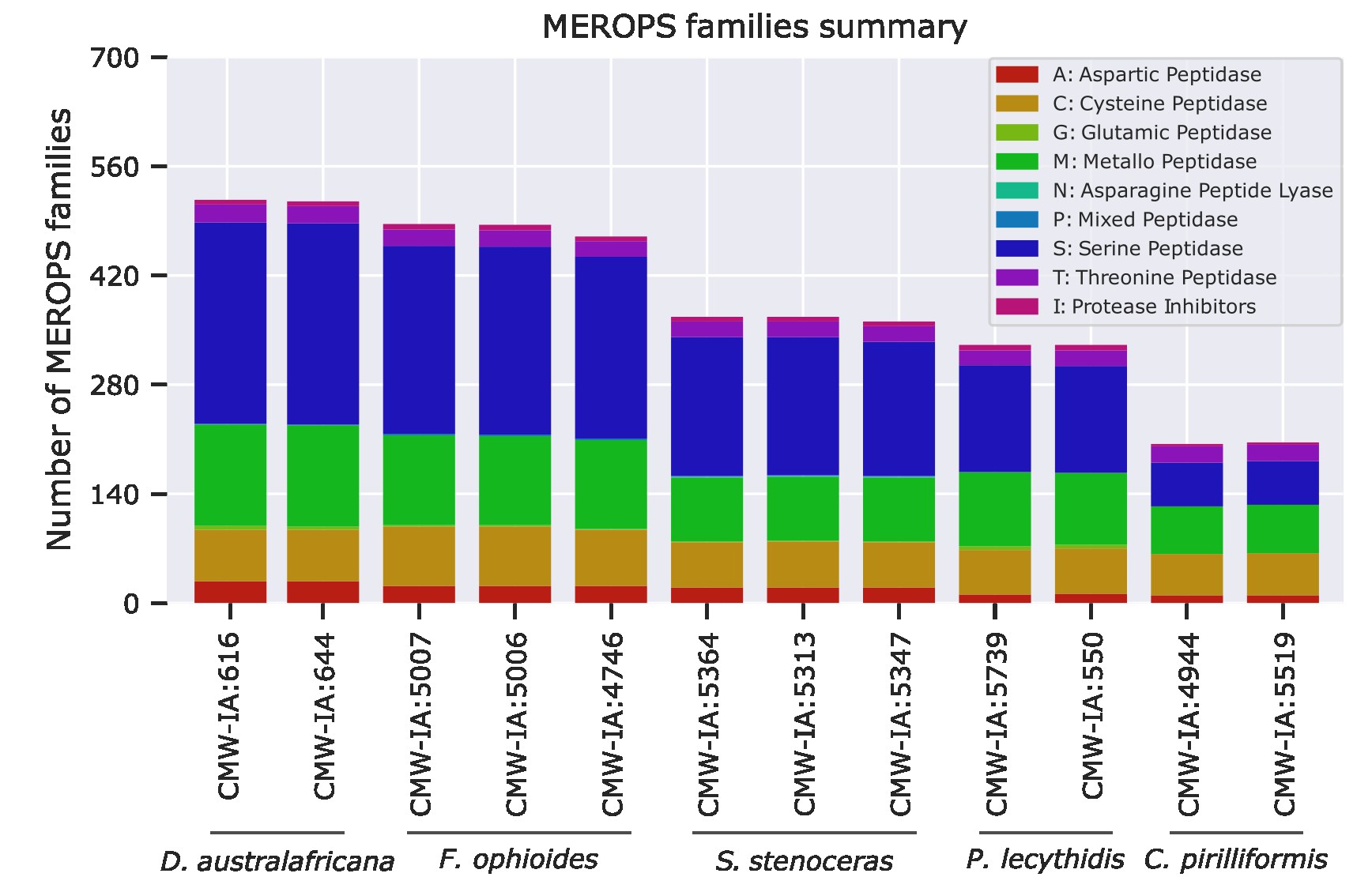


**Figure S2-3.** The number and types of peptidase families (identified via the MEROPs database) identified in the predicted proteomes of each strain.


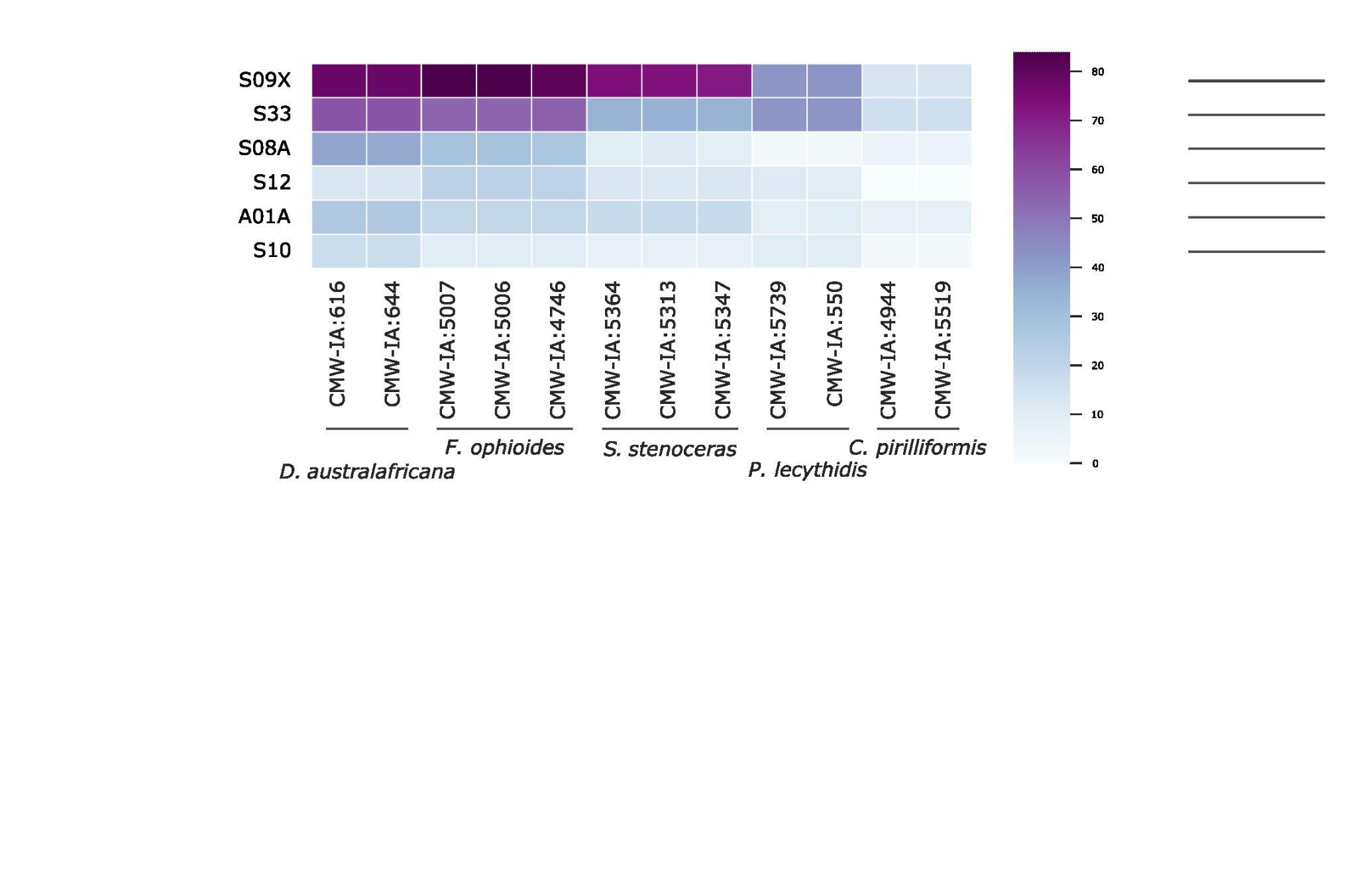


**Figure S2-4.** Heatmap illustrating the differences in the number of peptidase families identified in the predicted proteomes of each strain. Only peptidase families with copy number variation exceeding a standard deviation of four are shown (in descending order).

# Conserved protein domains


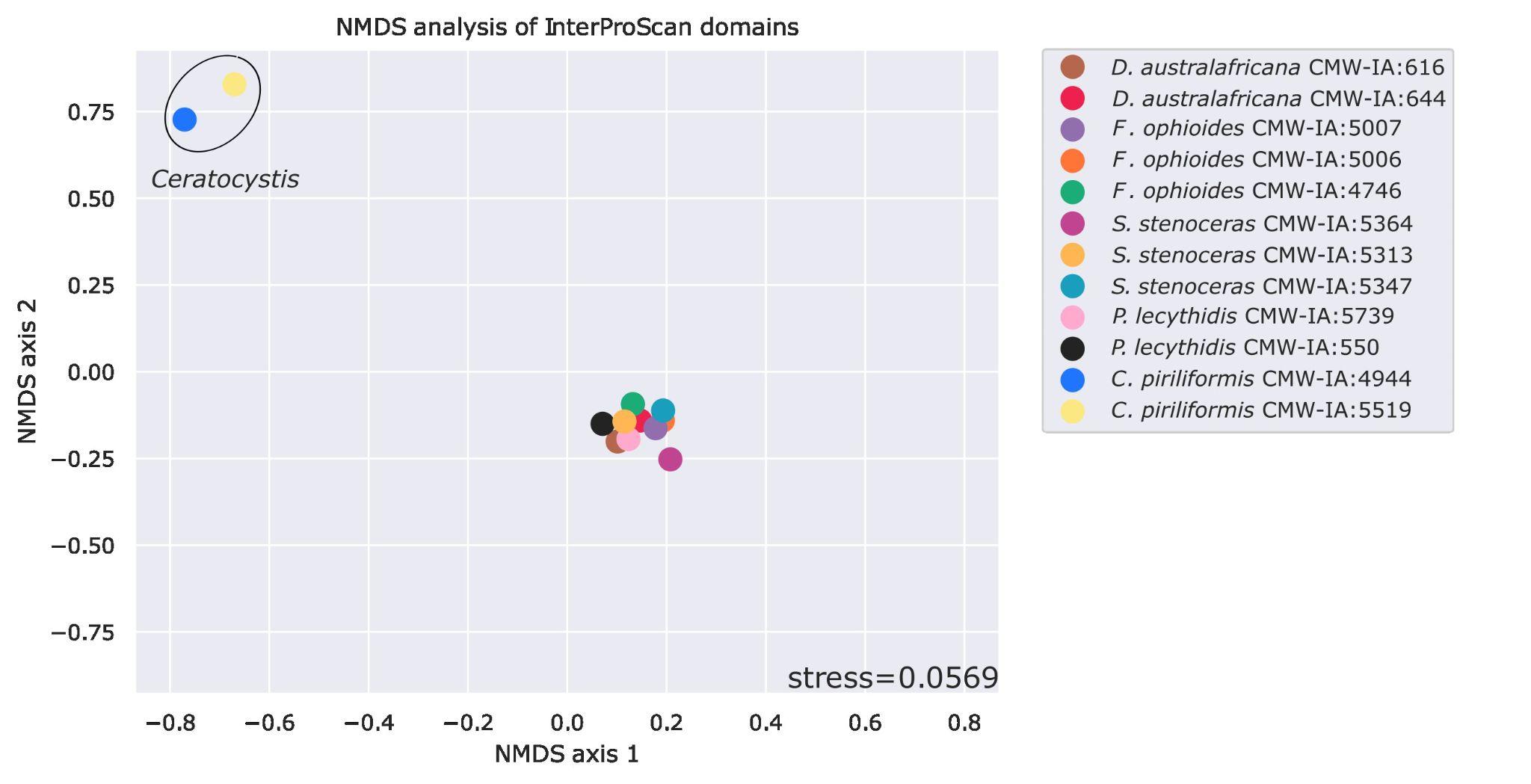


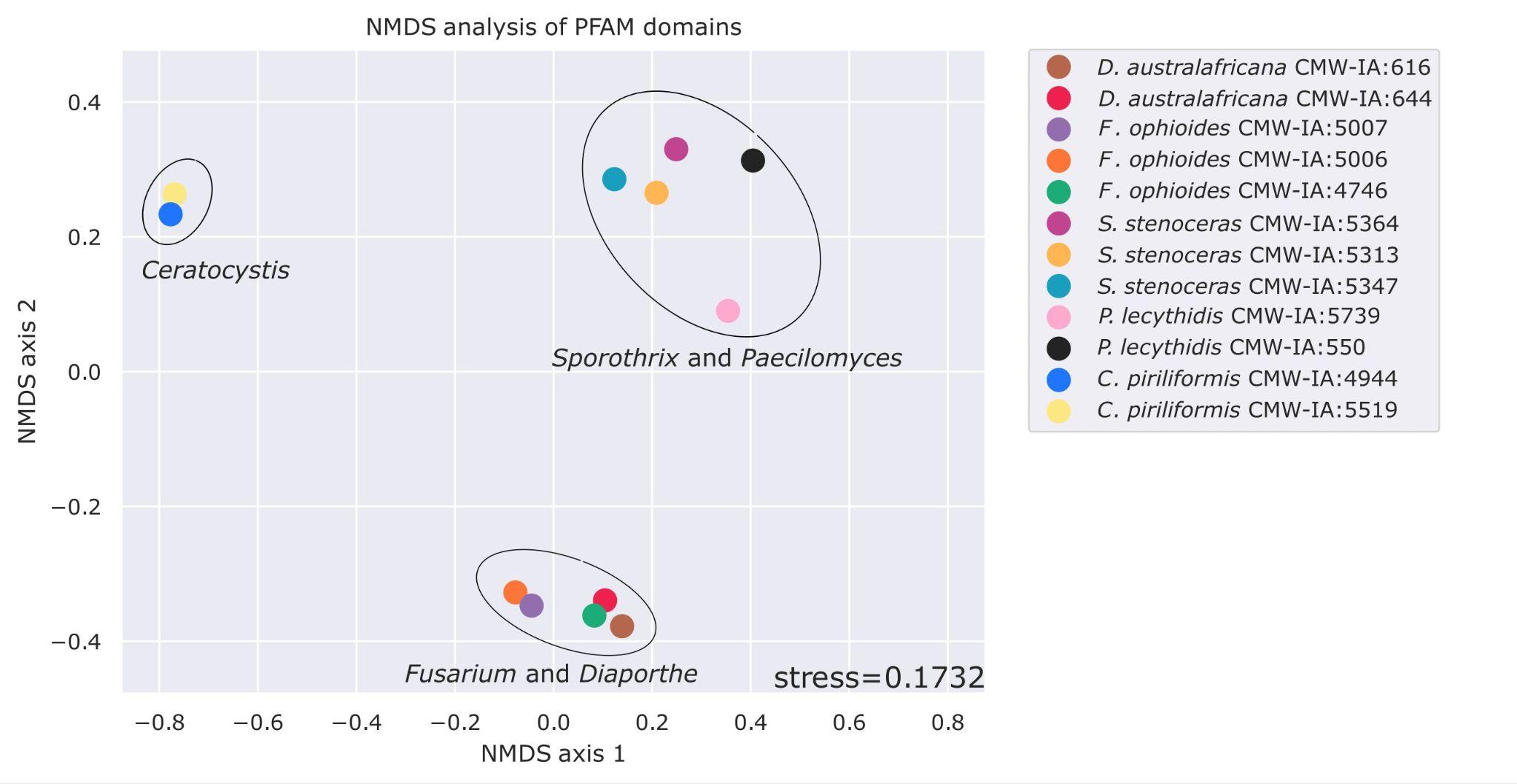


**Figure S2-5**. Nonmetric multidimensional scaling (NMDS) analysis to compare differences among the (a) InterProScan and (b) PFAM domains identified in the predicted proteomes of each strain.

# Transcription Factors


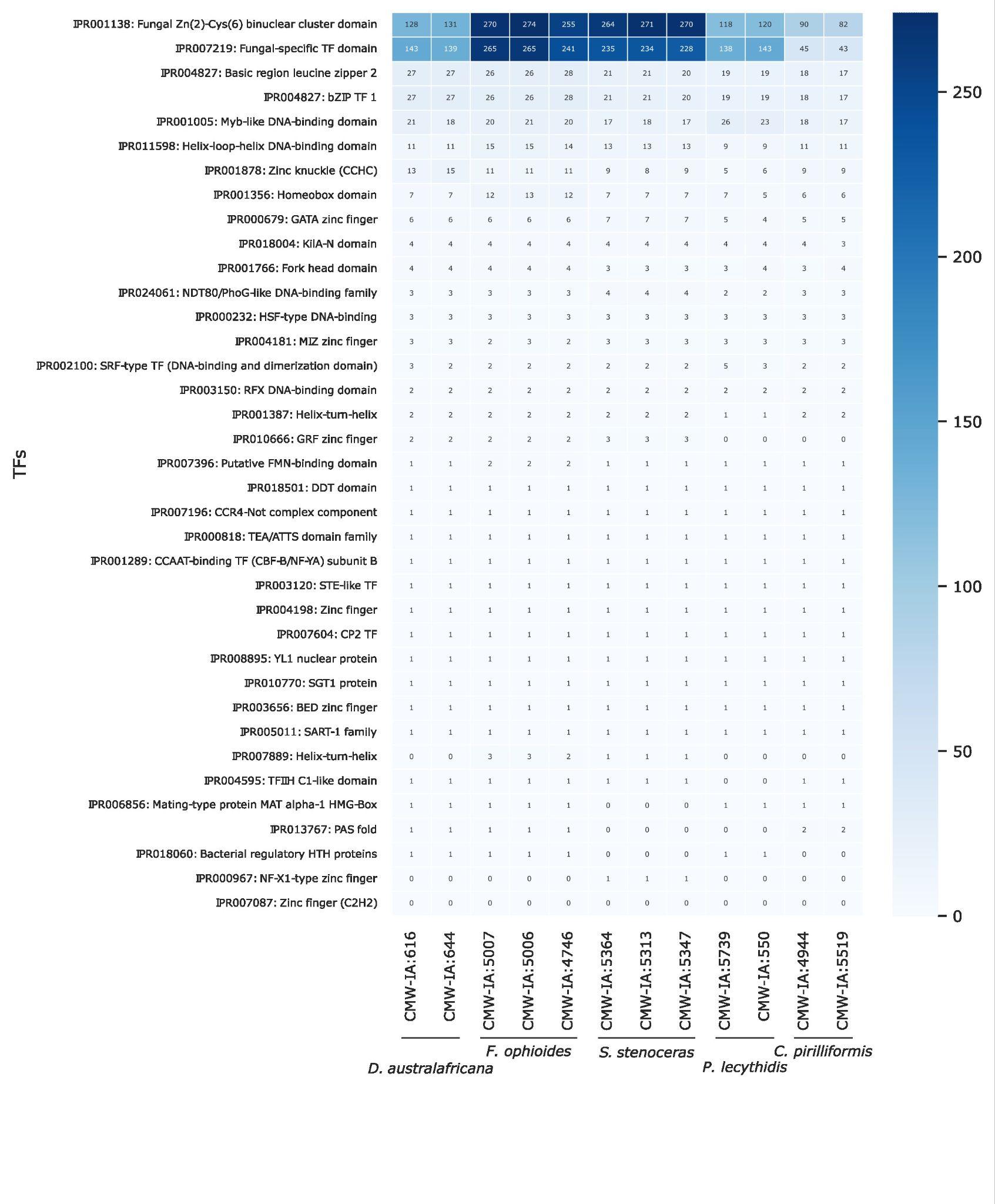


**Figure S2-6**. Transcription Factor InterProScan domains and their copy numbers identified in the predicted proteomes of each strain. Domains are presented in order of the average most abundant to the least abundant for this specific group of strains.
