# Supplementary material for: IMA Genome – F19: A genome assembly and annotation guide to empower mycologists, including annotated draft genome sequences of Ceratocystis pirilliformis, Diaporthe australafricana, Fusarium ophioides, Paecilomyces lecythidis, and Sporothrix stenoceras
Source: IMA Fungus. 2024 Jun 3;15:12. doi: 10.1186/s43008-024-00142-z (PMC11149380; doi:10.1186/s43008-024-00142-z)
Supplement: Supplementary file 5 — Supplementary Material 5. [file 43008_2024_142_MOESM5_ESM.pdf]

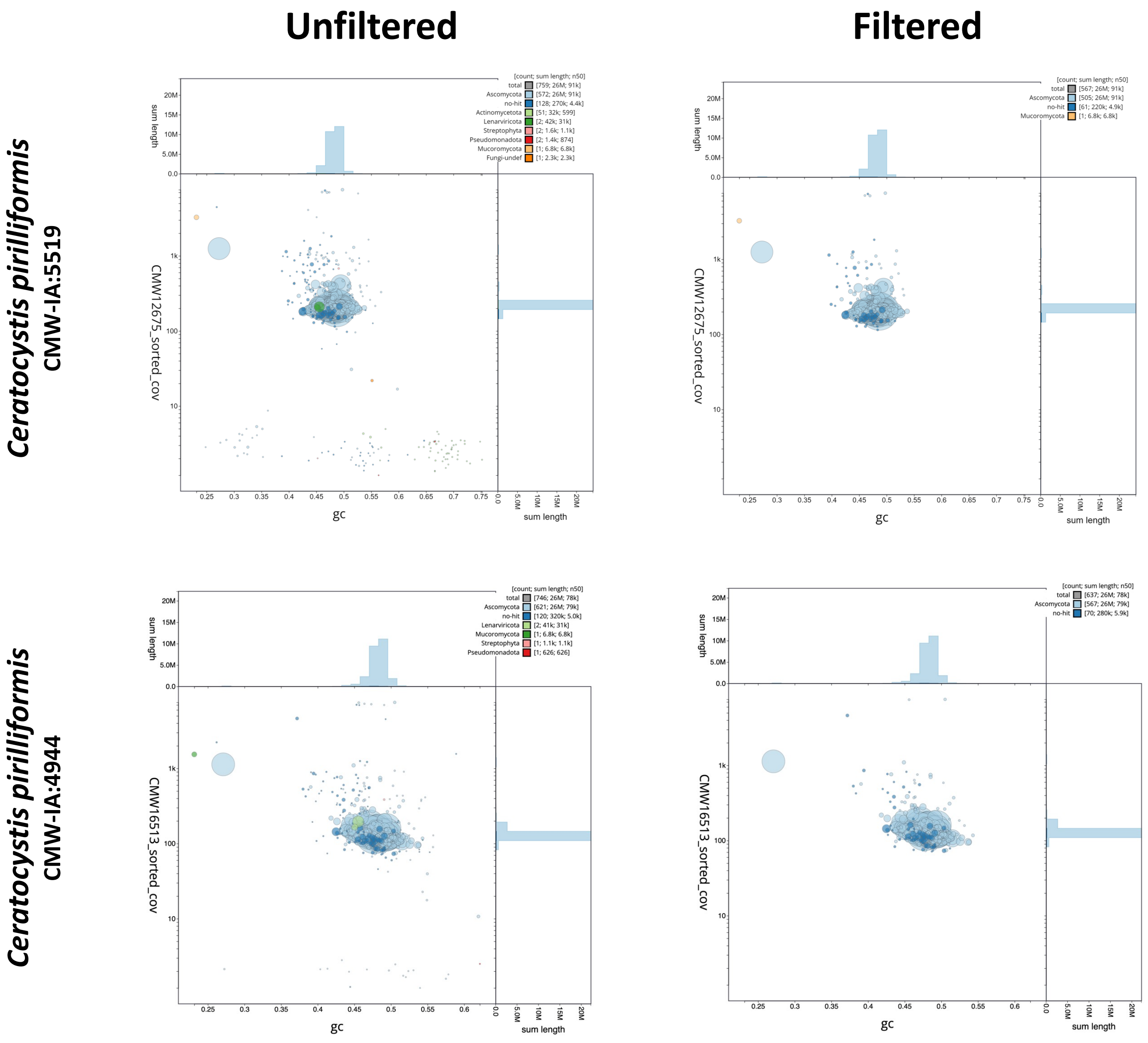

Fig. S1 Taxon-annotated GC-coverage plots (BlobPlots) for the two *Ceratocystis pirilliformis* genomes (CMW-IA:5519 and CMW-IA:4944). Both the unfiltered (left) and filtered (right) genomes are shown. The initial assemblies (v1.1) were filtered to keep contigs ≥1 000 bp in length and with at least 100X (CMW-IA:5519) or 67X (CMW-IA:4944) coverage depth.

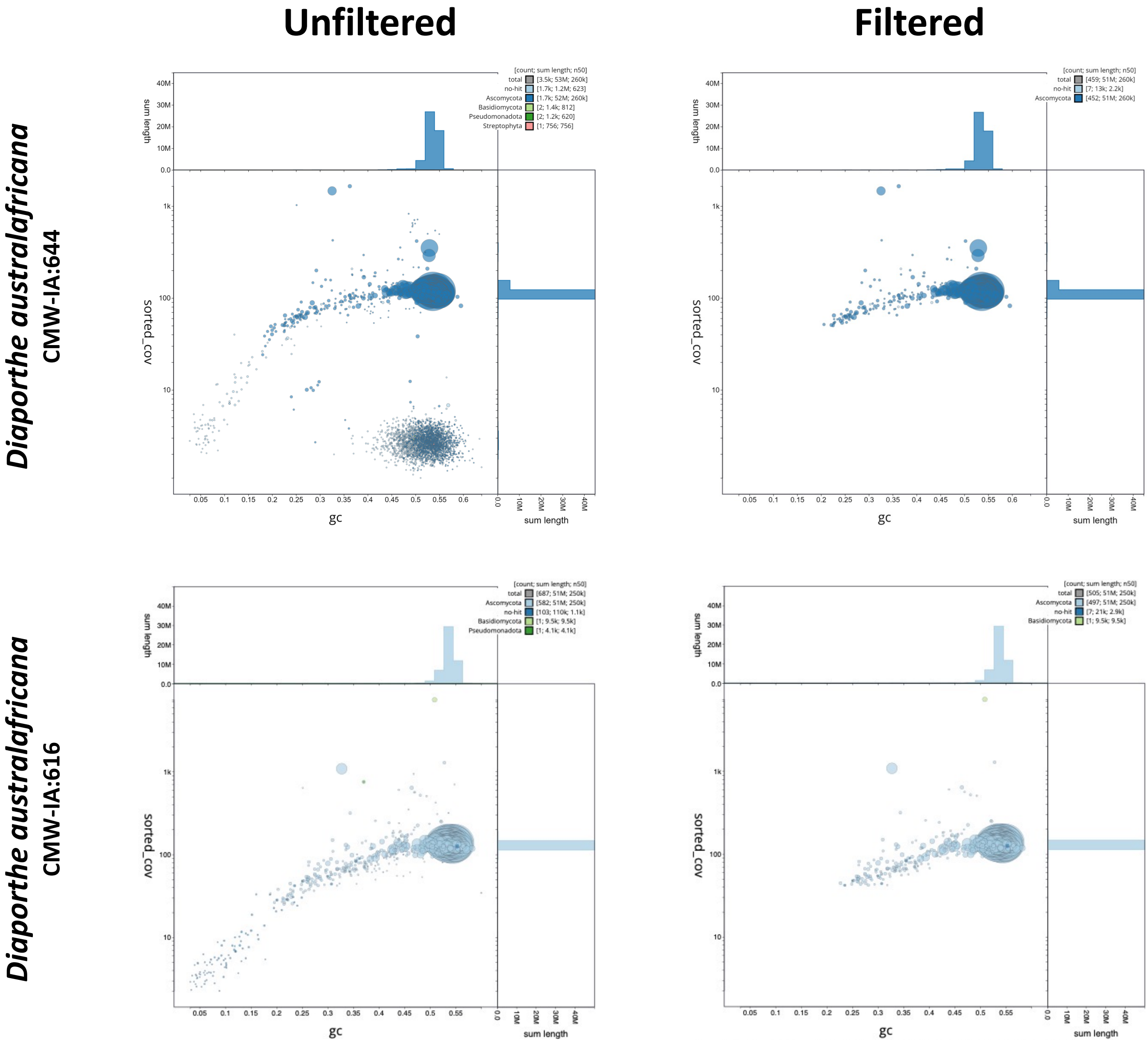

Fig. S2 Taxon-annotated GC-coverage plots (BlobPlots) for the two *Diaporthe australafricana* genomes (CMW-IA:644 and CMW-IA:616). Both the unfiltered (left) and filtered (right) genomes are shown. The initial assemblies (v1.1) were filtered to keep contigs ≥1 000 bp in length and with at least 15X coverage depth.

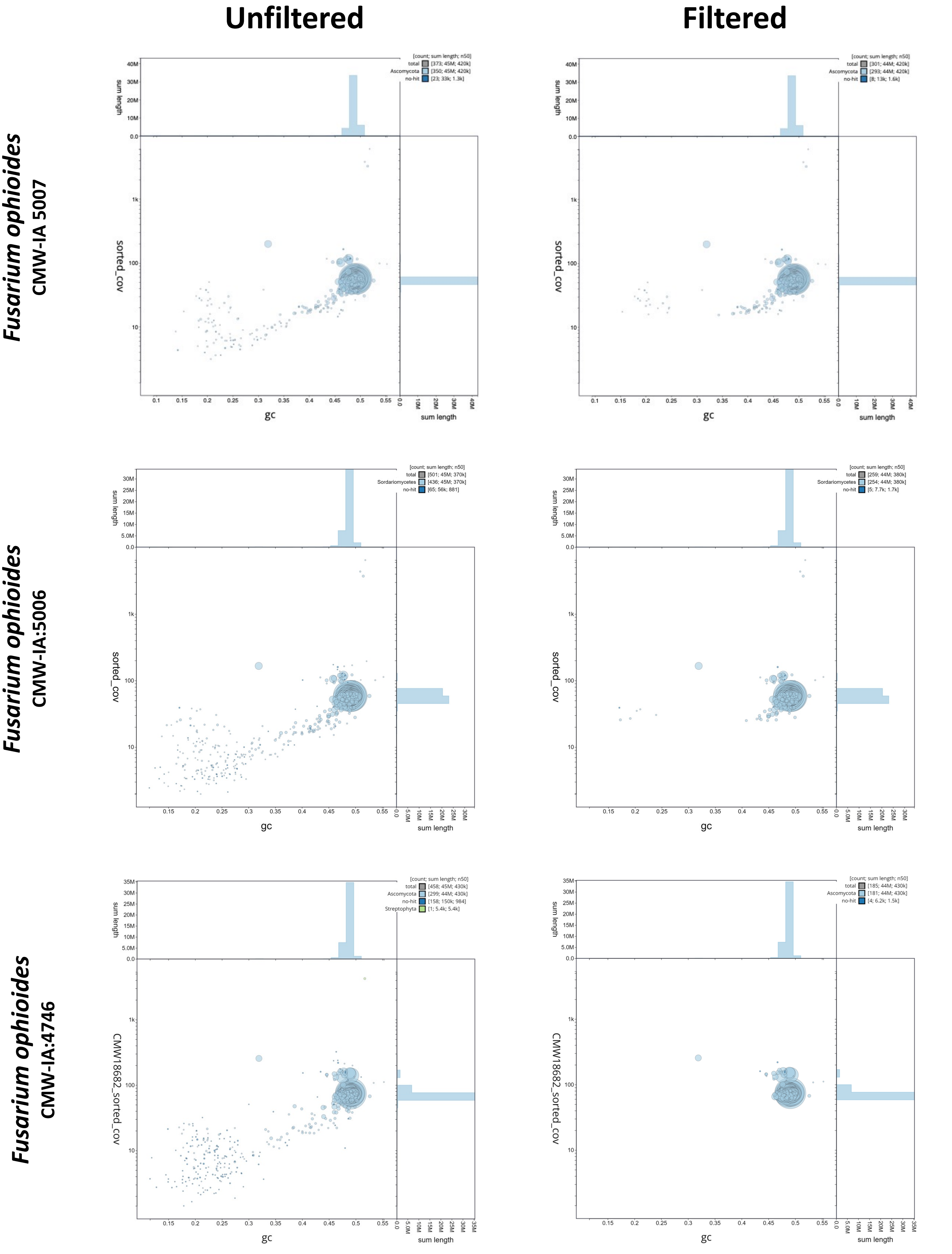

Fig. S3 Taxon-annotated GC-coverage plots (BlobPlots) for the three *Fusarium ophioides* genomes (CMW-IA:5007, CMW-IA:5006, and CMW-IA:4746). Both the unfiltered (left) and filtered (right) genomes are shown. The initial assemblies (v1.1) were filtered to keep contigs ≥1 000 bp in length and with at least 25X coverage depth.

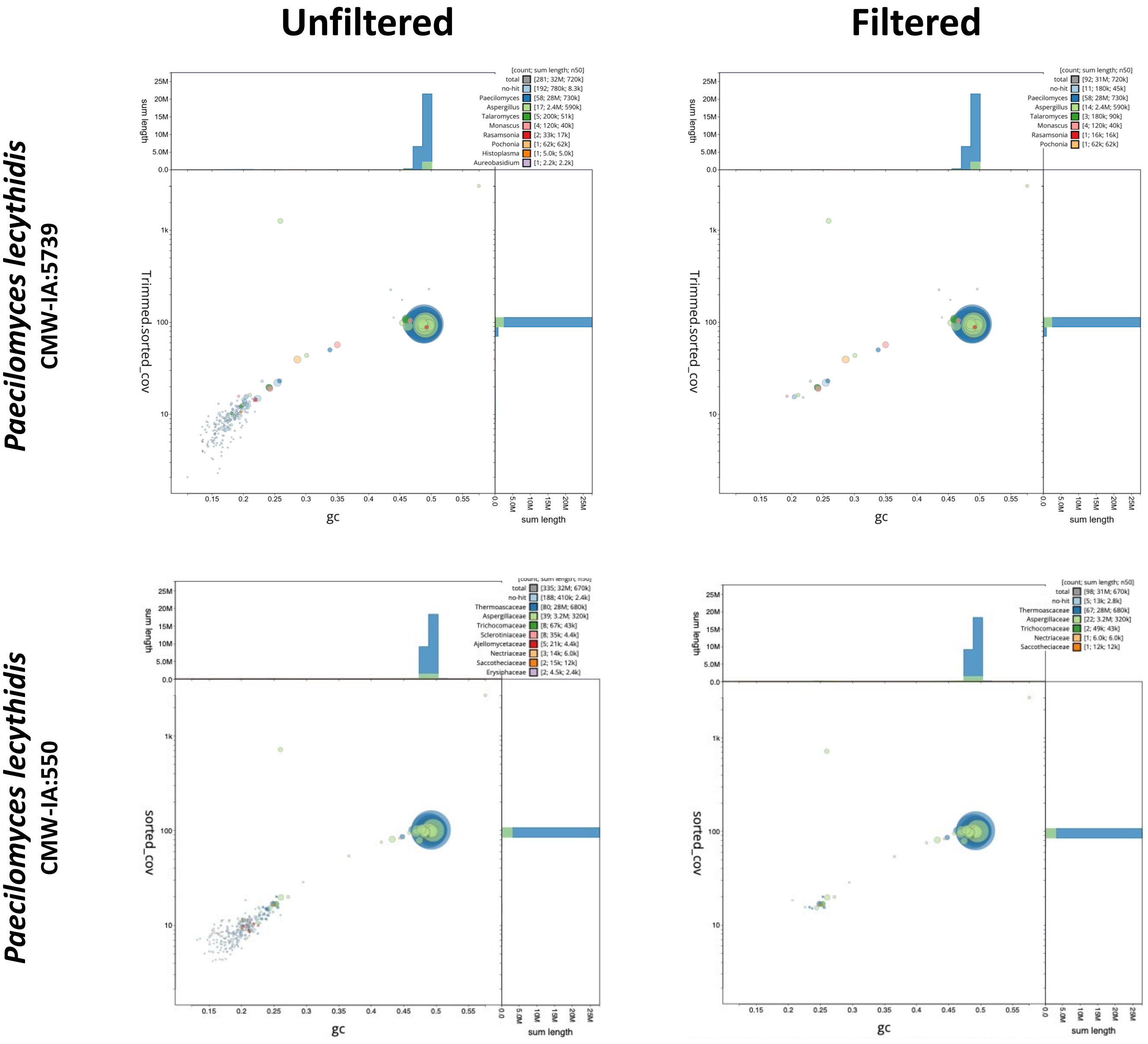

Fig. S4 Taxon-annotated GC-coverage plots (BlobPlots) for the two *Paecilomyces lecythidis* genomes (CMW-IA:5739 and CMW-IA:550). Both the unfiltered (left) and filtered (right) genomes are shown. The initial assemblies (v1.1) were filtered to keep contigs ≥1 000 bp in length and with at least 15X coverage depth.

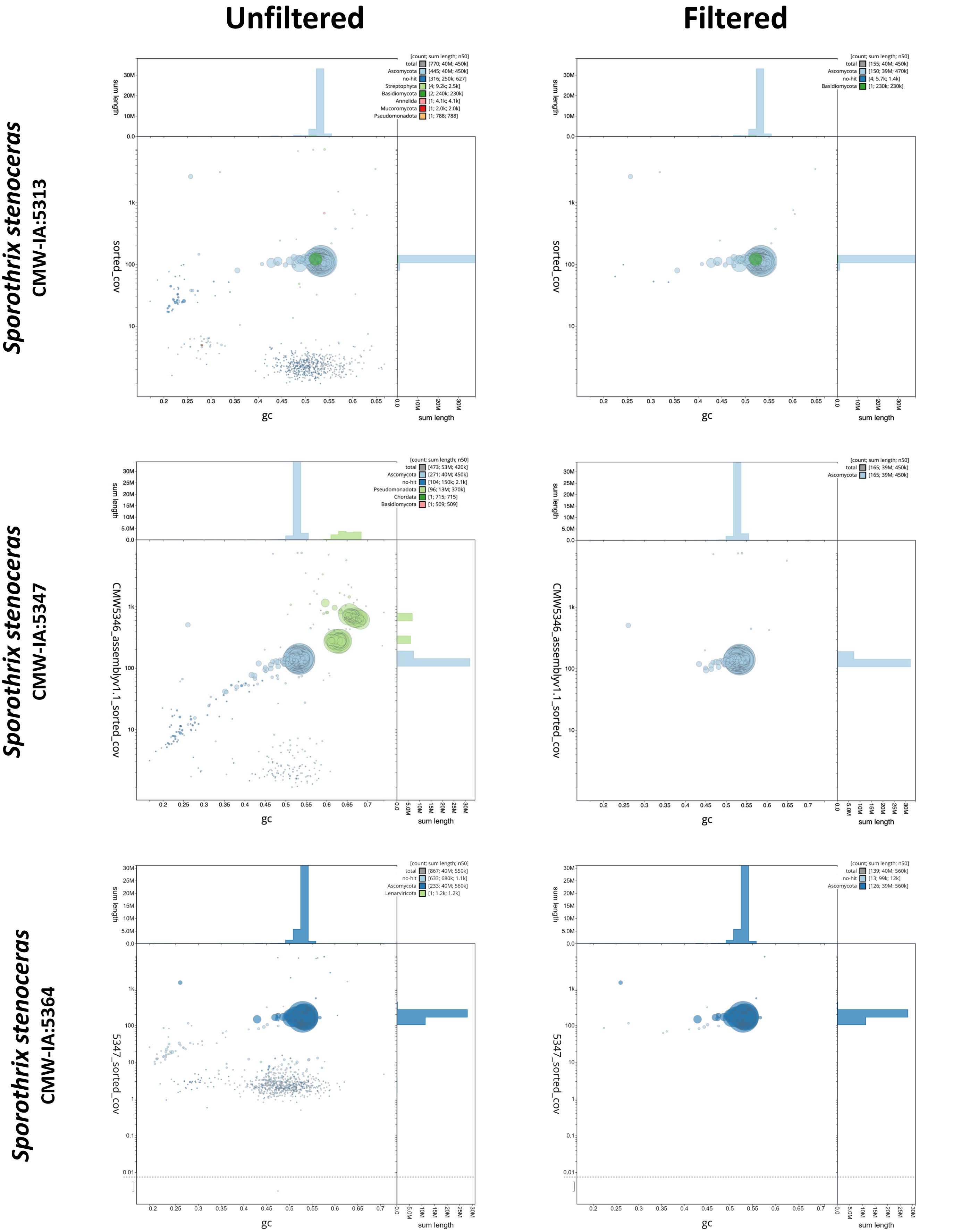

Fig. S5 Taxon-annotated GC-coverage plots (BlobPlots) for the three *Sporothrix stenoceras* genomes (CMW-IA:5313, CMW-IA:5347, and CMW-IA:5364). Both the unfiltered (left) and filtered (right) genomes are shown. The initial assemblies (v1.1) were filtered to keep contigs ≥1 000 bp in length and with at least 50X coverage depth. The assembly for CMW-IA:5347 was also filtered based on taxonomy, removing contigs labelled as *Pseudomonadota*, a bacterial phylum.
